# Ror2-mediated non-canonical Wnt signaling regulates Cdc42 and cell proliferation during tooth root development

**DOI:** 10.1242/dev.196360

**Published:** 2021-01-21

**Authors:** Yuanyuan Ma, Junjun Jing, Jifan Feng, Yuan Yuan, Quan Wen, Xia Han, Jinzhi He, Shuo Chen, Thach-Vu Ho, Yang Chai

**Affiliations:** 1 Center for Craniofacial Molecular Biology, University of Southern California, Los Angeles, CA 90033, USA; 2 Guangdong Provincial Key Laboratory of Stomatology, Department of Prosthodontics, Guanghua School of Stomatology, Hospital of Stomatology, Sun Yat-sen University, Guangzhou, 510055, PR China

**Keywords:** Ror2, Tooth root, Cell proliferation, Cdc42

## Abstract

The control of size and shape is an important part of regulatory process during organogenesis. Tooth formation is a highly complex process that fine-tunes the size and shape of the tooth, which are crucial for its physiological functions. Each tooth consists of a crown and one or more roots. Despite comprehensive knowledge of the mechanism that regulates early tooth crown development, we have limited understanding of the mechanism regulating root patterning and size during development. Here, we show that Ror2-mediated non-canonical Wnt signaling in the dental mesenchyme plays a crucial role in cell proliferation, and thereby regulates root development size in mouse molars. Furthermore, Cdc42 acts as a potential downstream mediator of Ror2 signaling in root formation. Importantly, activation of Cdc42 can restore cell proliferation and partially rescue the root development size defects in *Ror2* mutant mice. Collectively, our findings provide novel insights into the function of Ror2-mediated non-canonical Wnt signaling in regulating tooth morphogenesis, and suggest potential avenues for dental tissue engineering.

## Abstract (precis)

**Summary:** The function of Ror2-mediated non-canonical Wnt signaling and its effect on Cdc42 activation is crucial in regulating progenitor cell proliferation, odontoblast differentiation and Hertwig's epithelial root sheath formation during tooth root morphogenesis.

## INTRODUCTION

The tooth serves as an important model for investigating the regulatory mechanism of organ morphogenesis. Anatomically, teeth can be divided into two parts: the crown and the root. The development of the crown starts prenatally with early tooth morphogenesis. After crown development is completed, root formation initiates and proceeds through a complex process influenced by Hertwig's epithelial root sheath (HERS), cranial neural crest (CNC)-derived mesenchyme and adjacent anatomical structures (Li et al., 2017). The distribution of proliferation, apoptosis and osteoclast activity in these tissues corresponds with morphological changes during root development (Matalová et al., 2015). To date, we know little about the molecular mechanism that regulates root development size postnatally (Balic and Thesleff, 2015; Li et al., 2017).

Canonical (β-catenin-dependent) Wnt signaling originating from dental mesenchyme and/or epithelium is known to play a crucial role in root patterning and development (Bae et al., 2015; Kim et al., 2013; Lohi et al., 2010; Yamashiro et al., 2007; Yang et al., 2013). Non-canonical (β-catenin-independent) Wnt signaling comprises several pathways that are required for tissue formation and homeostasis (Patel et al., 2019). However, the function of non-canonical Wnt pathways during root development remains unknown.

Receptor tyrosine kinase (RTK)-like orphan receptor 2 (Ror2) is one of the non-canonical Wnt receptors. Ror2 plays significant roles during early embryonic development in several types of tissues, and Ror2 is a potential therapeutic target in cancer due to its association with cancer formation (Debebe and Rathmell, 2015). Ror2 can bind to Wnt5a to contribute to cytoskeletal arrangement, cell migration, cell polarity and cell proliferation, and to modulate downstream gene expression (He et al., 2008; Oishi et al., 2003; Schambony and Wedlich, 2007). Notably, Ror2 can inhibit or activate canonical Wnt signaling at the level of TCF/LEF–mediated transcription depending on whether Wnt ligands are present (Billiard et al., 2005; Mikels and Nusse, 2006). Owing to these interesting features, including its significance in development and potential for cancer therapy, Ror2 has attracted considerable attention.

In humans, *ROR2* mutations are associated with diseases such as dominant Brachydactyly B (BDB, a shortening of the digits) (Oldridge et al., 2000) and recessive Robinow syndrome (RRS), which is associated with short-limbed dwarfism, abnormal vertebrae, fusion of the ribs and abnormal facial structures (Afzal and Jeffery, 2003). Interestingly, some individuals with RRS also have dental problems, such as fusion of primary teeth, delayed eruption of the permanent teeth and delayed root formation of the permanent teeth, suggesting the involvement of ROR2 in tooth development (Jain et al., 2017). In mice, *Ror2* is expressed in the dental epithelium and mesenchyme at embryonic stages (Lin et al., 2011; Schwabe et al., 2004). *Ror2*^−/−^ mice exhibit retarded crown formation and defective odontoblast differentiation, which are also observed in Wnt5a-deficient mice (Lin et al., 2011). This indicates that Ror2 may participate in mediating Wnt5a signaling during early tooth development. However, roles of ROR2/Ror2 are tissue-specific (Roarty et al., 2017; Sun et al., 2015). Therefore, the issues of whether Ror2 expressed in specific tissues has the capacity to regulate tooth development, and whether it modulates root formation, remain unclear.

In the present study, we show that crown formation is unaffected in *Ror2* conditional knockout mouse models but loss of *Ror2* in the dental mesenchyme leads to delayed root elongation and ultimately to shortened roots. We further establish that mesenchymal Ror2 regulates cell proliferation, odontoblast differentiation in the apical region of the tooth, and HERS formation through tissue-tissue interactions during postnatal development. Furthermore, we have identified cell division cycle 42 (Cdc42) as a downstream mediator of Ror2 signaling in the regulation of cell proliferation during root development. Importantly, activation of Cdc42 can partially rescue the root development size defect in *Ror2* mutant mice, clearly demonstrating the functional significance of Ror2 and its impact on Cdc42 activation in regulating root development postnatally.

## RESULTS

### Ror2 in the dental mesenchyme plays a crucial role in mouse molar root morphogenesis

**Fig. 1.**
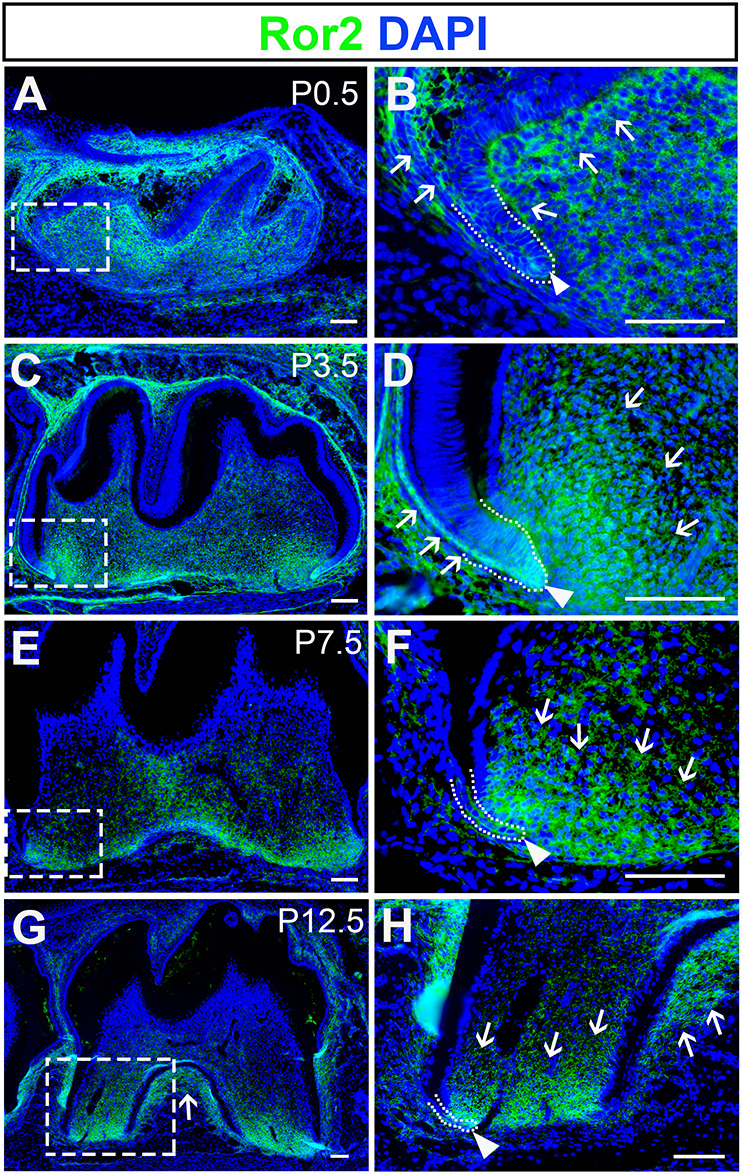
**Ror2 is expressed in the dental mesenchyme and epithelium during tooth root development.** (A-H) Ror2 immunofluorescence (green) of sagittal sections of mandibular molars from wild-type mice at indicated stages from P0.5 to P12.5. Boxes in A, C, E and G are enlarged in B, D, F and H, respectively. Dotted lines indicate the border between dental epithelium and dental mesenchyme. Arrowheads indicate dental epithelium, and arrows indicate localization of Ror2 expression. Scale bars: 100 µm.

Tooth root development begins at around postnatal day (P)3.5 and completes when the tooth erupts at around P18.5 (Li et al., 2017). To investigate the function of Ror2 during molar root development, we investigated its expression pattern at different root development stages. At P0.5, Ror2 expression was widely distributed in the dental mesenchyme and dental epithelium of wild-type mice (Fig. 1A,B) and gradually became more prominent apically in the root-forming region (Fig. 1C-F). The expression of Ror2 was also detectable in the periodontal region and alveolar bone at P12.5 (Fig. 1G,H). As the apical dental mesenchyme is closely associated with tooth growth, this enriched apical expression suggests that Ror2 may play a specific role in root development.

To establish the functional significance of Ror2-mediated signaling in tooth development, we generated *Osr2-Cre;Ror2^fl/fl^* mice with specific ablation of Ror2 in the dental mesenchyme. The expression of Osr2 is detectable in the craniofacial region from embryonic day (E)10.5 (Lan et al., 2007) and in the dental mesenchyme from E12.5 (Li et al., 2011). Throughout tooth development, the *Osr2-Cre* transgene targets the dental mesenchyme and the alveolar bone, but not the dental epithelium, in a lineage tracing study (Fig. S1A-D). Ror2 expression was undetectable in the dental mesenchyme but still present in the epithelium of *Osr2-Cre;Ror2^fl/fl^* mice at P3.5 and P9.5 (Fig. S1E-H). This expression pattern indicated highly efficient mesenchyme-specific deletion of *Ror2* in *Osr2-Cre;Ror2^fl/fl^* mice during tooth morphogenesis.

The morphology of the molar tooth crowns in *Osr2-Cre;Ror2^fl/fl^* mice was similar to that of control molars at P0.5 and P2.5 (Fig. S2A-J), before root development. We observed no significant differences in cell proliferation or differentiation when comparing the crowns of control and *Osr2-Cre;Ror2^fl/fl^* molars during this period based on Ki67 immunostaining (Fig. S2K,L), RNAscope *in situ* hybridization of *Dspp* and EdU assay (Fig. S2M,N). Therefore, we concluded that crown patterning is unaffected following the loss of *Ror2* in the dental mesenchyme in *Osr2-Cre;Ror2^fl/fl^* mice.

**Fig. 2.**
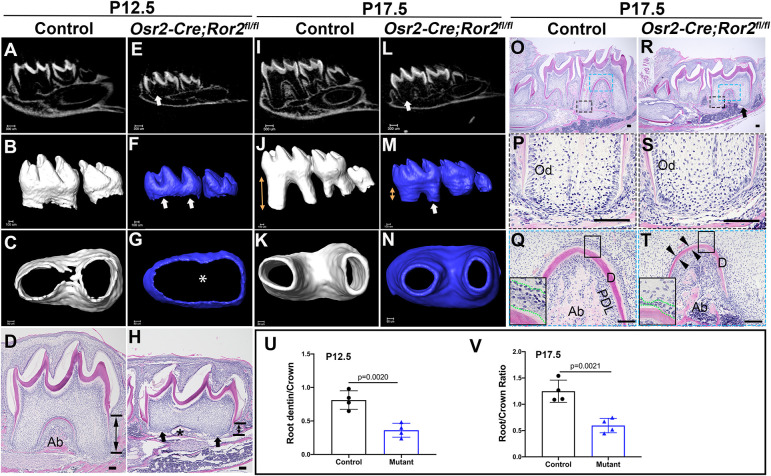
**Loss of *Ror2* in the dental mesenchyme results in root development defects.** (A-H) MicroCT images and H&E staining of control (A-D) and *Osr2-Cre;Ror2^fl/fl^* (E-H) mandibular molars at P12.5. Arrows indicate the apical region and asterisks indicate furcation region. Double-headed arrows in D and H indicate root dentin. *n*=4. (I-N) MicroCT images of control (I-K) and *Osr2-Cre;Ror2^fl/fl^* (L-N) mandibular molars at P17.5. Arrows indicate the apical region of the root and double-headed arrows indicate the length of the root. *n*=4. (O-T) H&E staining of control (O-Q) and *Osr2-Cre;Ror2^fl/fl^* (R-T) mandibular molars at P17.5. Boxes in O are enlarged in P and Q; boxes in R are enlarged in S and T. Arrows indicate the apical region of the root, and arrowheads indicate the dentin in the furcation region. Boxes in Q and T are shown in the insets at higher magnification to reveal the morphology of odontoblasts. (U,V) Quantification analysis of the ratio of root to crown in control and *Osr2-Cre;Ror2^fl/fl^* (mutant) mice at P12.5 and P17.5 (*n*=4; unpaired, two-tailed Student's *t*-test). Data are mean±s.e.m. AB, alveolar bone; D, dentin; Od, odontoblast; PDL, periodontal ligament. Scale bars: 100 µm (D,H,O-T).

We then further compared the tooth root formation of control and *Osr2-Cre;Ror2^fl/fl^* mice. At P12.5, the root elongation and furcation formation processes had commenced in the mandibular first molars of control mice (*n*=4; Fig. 2A-D). However, root elongation was delayed and no furcation had formed in *Osr2-Cre;Ror2^fl/fl^* molars at this stage (*n*=4; Fig. 2E-H,U). By P17.5, the loss of *Ror2* in the dental mesenchyme had resulted in shortened roots, but a normal root number, in mandibular first molars in *Osr2-Cre;Ror2^fl/fl^* mice (*n*=4; Fig. 2I-T,V). Histological analysis showed that dentin and alveolar bone formation in the furcation region were disrupted in *Osr2-Cre;Ror2^fl/fl^* mice compared with controls (Fig. 2Q,T). Additionally, the odontoblasts in the furcation region were shorter in *Ror2* mutant molars than in controls (Fig. 2Q,T). Furthermore, the mandible was smaller in *Osr2-Cre;Ror2^fl/fl^* mice compared with controls (Fig. 2A,E,I,L,O,R). In order to determine whether the root size defect was caused by loss of *Ror2* in the dental mesenchyme or was secondary to the mandibular bone defect, we collected tooth germs of the mandibular first molars at P4.5 from *Osr2-Cre;Ror2^fl/fl^* and control mice, and transplanted them into the kidney capsule. After 3 weeks of cultivation, we observed that the *Osr2-Cre;Ror2^fl/fl^* molars had shortened roots with normal crown formation (*n*=3; Fig. S3), which is consistent with the phenotype observed *in vivo* and suggests that the root size defect is not secondary to the mandibular defect in *Osr2-Cre;Ror2^fl/fl^* mice.

### Ablation of *Ror2* in the dental mesenchyme disrupts mesenchymal cell proliferation and differentiation

**Fig. 3.**
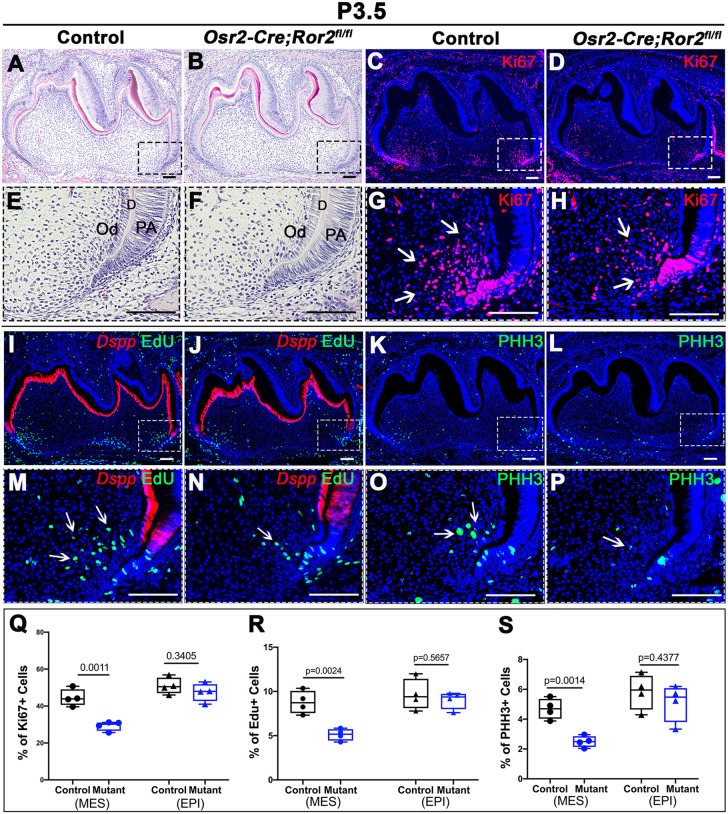
**Loss of *Ror2* in dental mesenchyme decreases mesenchymal cell proliferation.** (A,B,E,F) H&E staining of control (A,E) and *Osr2-Cre;Ror2^fl/fl^* (B,F) mandibular molars at P3.5. Boxes in A and B are enlarged in E and F, respectively. (C,D,G,H) Ki67 immunofluorescence (red) indicating proliferating cells in sagittal sections of mandibular molars in control (C,G) and *Osr2-Cre;Ror2^fl/fl^* (D,H) mandibular molars at P3.5. Boxes in C and D are enlarged in G and H, respectively. Arrows indicate positive signals. (I,J,M,N) RNAscope *in situ* hybridization of *Dspp* (red) and EdU assay (green) in control (I,M) and *Osr2-Cre;Ror2^fl/fl^* (J,N) mandibular molars at P3.5 2 h after EdU injection. Boxes in I and J are enlarged in M and N, respectively. Arrows indicate positive signals. (K,L,O,P) PHH3 immunofluorescence (green) indicating mitotic cells in control (K,O) and *Osr2-Cre;Ror2^fl/fl^* (L,P) mandibular molars at P3.5. Boxes in K and L are enlarged in O and P, respectively. Arrows indicate positive signals. (Q-S) Quantification of Ki67^+^ cells (Q), EdU-labeled cells (R) and PHH3^+^ cells (S) in dental mesenchyme and dental epithelium in the apical region shown in G, H, M, N, O and P (*n*=4; unpaired, two-tailed Student's *t*-test). Data are mean±s.e.m. D, dentin; EPI, epithelium; MES, mesenchyme; Od, odontoblast; PA, pre-ameloblast. Scale bars: 100 µm.

We next evaluated the cellular proliferation activity in the apical region during root development. At P3.5, there was no obvious difference in the tissue morphology in the apical region between control and *Ror2* mutant molars (Fig. 3A,B,E,F). However, a significant decrease in cell proliferation in the dental mesenchyme of *Osr2-Cre;Ror2^fl/fl^* molars was observed as we compared Ki67^+^ (actively cycling) cells in control and *Ror2* mutant mice at P3.5 (Fig. 3C,D,G,H,Q). Despite this, the proliferation in the root epithelium was not significantly different between *Osr2-Cre;Ror2^fl/fl^* and control mice (Fig. 3Q). Consistent with this hypoproliferative cell phenotype in the mesenchyme, we detected significantly reduced expression of S- and M-phase cell cycle markers, suggesting that cell cycle progression was inhibited in *Osr2-Cre;Ror2^fl/fl^* molars compared with controls (Fig. 3I-P,R,S). Two hours after EdU injection, the number of EdU-labeled cells conducting cDNA synthesis (S phase) was significantly decreased in the dental mesenchyme of *Osr2-Cre;Ror2^fl/fl^* mice at P3.5 (Fig. 3I,J,M,N,R). The expression of PHH3, a marker of mitotic cells, was also reduced in the dental mesenchyme of *Osr2-Cre;Ror2^fl/fl^* mice (Fig. 3K,L,O,P,S). However, the numbers of EdU-labeled and PHH3-expressing cells were normal in the dental epithelium of *Osr2-Cre;Ror2^fl/fl^* mice (Fig. 3R,S). Additionally, there was no difference in apoptotic activity in the dental mesenchyme of *Osr2-Cre;Ror2^fl/fl^* or control mice at P3.5, P5.5 and P9.5 (Fig. S4). This suggests that Ror2 may be required for normal proliferation of dental mesenchymal cells during root development, but not for apoptosis.

A previous study suggested that dental mesenchyme proliferation is crucial for root elongation and for guiding HERS formation during root development (Sohn et al., 2014). We thus investigated the formation of HERS. Morphological changes in HERS were first observed at P5.5 (Fig. S5A-D), 2 days later than the cellular defects in the dental mesenchyme of *Osr2-Cre;Ror2^fl/fl^* mice (Fig. 3). It was particularly obvious that HERS invagination formed a thicker and shorter structure in *Osr2-Cre;Ror2^fl/fl^* molars at P7.5 compared with the normal bi-layered structure in the controls (Fig. S5E,F). At P9.5, HERS lost continuity in both control and *Ror2* mutant molars; in *Ror2* mutant mice, HERS formed a structure that was similar to but shorter than what was seen in the controls (Fig. S5G,H). These findings suggest that loss of *Ror2* in the dental mesenchyme affects the development of HERS through tissue-tissue interaction.

**Fig. 4.**
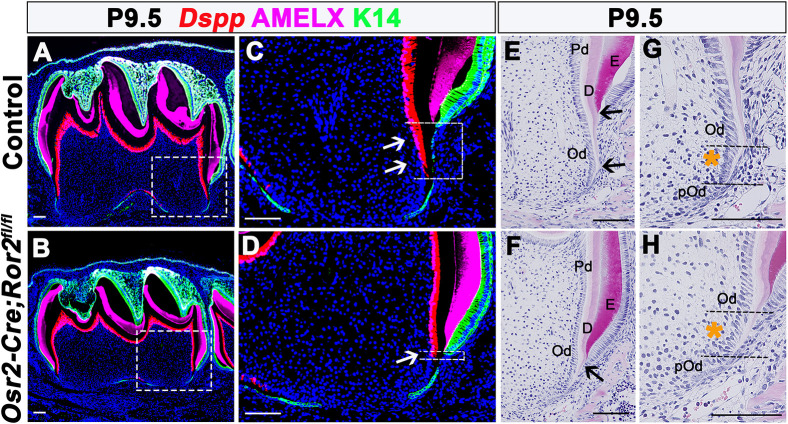
**Loss of *Ror2* in dental mesenchyme disrupts differentiation of odontoblasts.** (A-D) RNAscope *in situ* hybridization of *Dspp* (red), and double immunostaining of amelogenin X (AMELX, purple) and K14 (green) represent odonblasts, enamel and dental epithelium, respectively, on sagittal sections of control (A,C) and *Osr2-Cre;Ror2^fl/fl^* (B,D) mandibular molars at P9.5. Boxes in A and B are shown at higher magnification in C and D, respectively. Arrows and dashed lines in C and D indicate *Dspp*^+^ cells below the enamel level. (E-H) Histological analysis of dentin formation (E,F) and morphology of odontoblasts (G,H) in the apical region from control and *Osr2-Cre;Ror2^fl/fl^* mice. Arrows indicate root dentin. Yellow asterisks between the dashed lines indicate the odontoblasts between differentiated odontoblasts and preodontoblasts. D, dentin; E, enamel; Od, odontoblast; pOd, preodontoblast; Pd, pre-dentin. Scale bars: 100 µm.

In addition, we investigated the expression of *Dspp*, a marker of terminally differentiated odontoblasts, in *Osr2-Cre;Ror2^fl/fl^* and control mice at P9.5. We found no differences in *Dspp* expression throughout the crown region (Fig. 4A,B). However, fewer *Dspp*^+^ cells were observed in the apical region below the enamel level in *Osr2-Cre;Ror2^fl/fl^* mice (Fig. 4C,D). Hemotoxylin and eosin (H&E) staining showed that dentinogenesis was retarded in the apical region in *Osr2-Cre;Ror2^fl/fl^* mice (Fig. 4E,F). There was no obvious change in the morphology of odontoblasts or preodontoblasts in *Ror2* mutant mice. In comparing them to a detailed published description of odontoblasts in the apical root region (Bae et al., 2013), we found that cells in the transition zone from pre-odontoblasts to odontoblasts showed a looser alignment in *Osr2-Cre;Ror2^fl/fl^* molars compared with a condensed columnar alignment in control molars (yellow asterisks; Fig. 4G,H). These results indicated that loss of *Ror2* in the dental mesenchyme disrupts differentiation of odontoblasts and dentinogenesis during root elongation, eventually resulting in the phenotypes observed at P12.5 and P17.5 (Fig. 2).

### Ror2 in the dental epithelium is not required for root development

Ror2 is expressed in the dental epithelium, as well as in the mesenchyme in mouse molars (Fig. 1). To investigate whether epithelial Ror2 is crucial for root formation, we generated *K14-rtTA;Teto-Cre;Ror2^fl/fl^* mice, in which *Ror2* is ablated in the dental epithelium at P3.5 (Fig. S6A-D). Based on microCT images and H&E staining, we found no significant differences in root length or root number between *K14-rtTA;Teto-Cre;Ror2^fl/fl^* and control mice at P21.5 (Fig. S6E-L). These findings indicated that epithelial Ror2 is not required for root formation.

### Activation of Cdc42 partially rescues root development and restores mesenchymal cell proliferation in *Ror2* mutant mice

It has been reported that Ror2 participates in both canonical and non-canonical Wnt signaling (Billiard et al., 2005; He et al., 2008). For example, Ror2 has been identified as a regulator of canonical Wnt signaling that affects the stabilization of cytosolic β-catenin *in vivo* and *in vitro* (Billiard et al., 2005; Mikels and Nusse, 2006; Roarty et al., 2017). Thus, we assessed the expression of *Axin2* and active β-catenin in the dental mesenchyme of the mandibular first molars. We found no significant differences between *Osr2-Cre;Ror2^fl/fl^* and control molars at P3.5 (Fig. S7A-D,I), which indicated that canonical Wnt signaling is not involved in the decreased cell proliferation caused by loss of *Ror2* in the dental mesenchyme. On the other hand, it has been shown that Ror2-mediated non-canonical Wnt signaling leads to transcriptional responses via c-Jun (also known as Jun) and ATF2 in *Xenopus* (Schambony and Wedlich, 2007). However, there were no distinguishable differences in the expression of phospho-c-Jun or phospho-ATF2 between *Osr2-Cre;Ror2^fl/fl^* and control molars at P3.5 (Fig. S7E-H). The activation of the small GTPase Rac1, an upstream mediator of c-Jun and ATF2, was also unchanged in the Ror2 mutant mice (Fig. S7J). Our results indicated that Ror2-mediated signaling did not trigger the activation of c-Jun and ATF2 in mouse molar root context. Owing to the complexity of non-canonical Wnt pathways and the context-dependent function of Ror2, we hypothesized that Ror2 may regulate tooth root development via other mediators of non-canonical Wnt signaling.

**Fig. 5.**
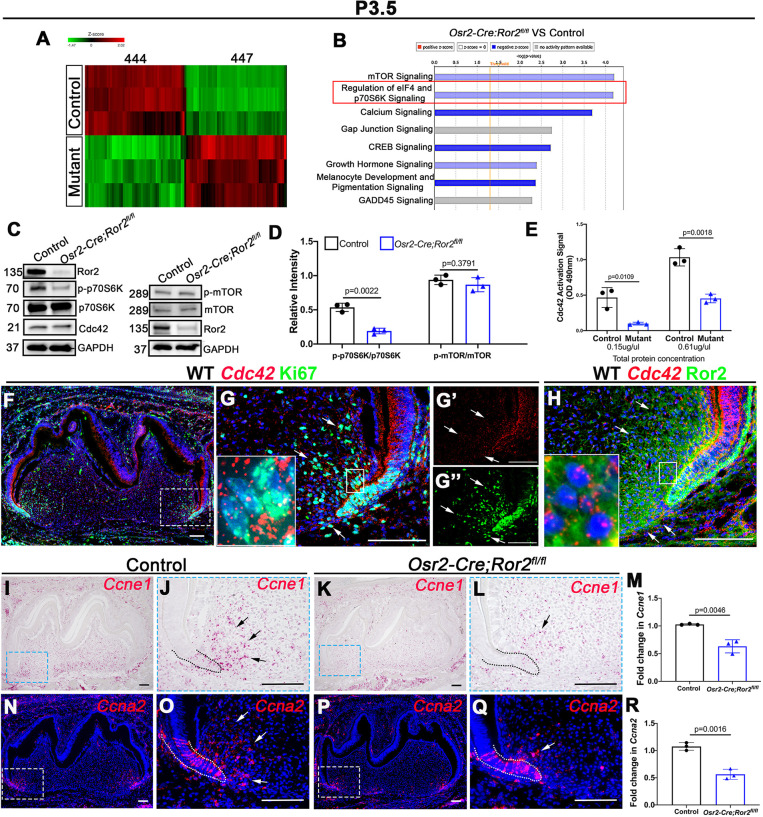
**Putative downstream targets after loss of *Ror2* in dental mesenchyme.** (A) Heat map showing the gene expression profiles of dental mesenchyme in control and *Osr2-Cre;Ror2^fl/fl^* (mutant) mandibular molars at P3.5. (B) Ingenuity pathway analysis based on RNA-seq data shows the downregulation of eIF4 and p70S6K signaling in *Osr2-Cre;Ror2^fl/fl^* mice (indicated by the red box). (C) Western blot of Ror2, Cdc42, p70S6K, phospho (p)-p70S6K, mTOR, p-mTOR and GAPDH in the dental mesenchyme from control and *Osr2-Cre;Ror2^fl/fl^* mice at P3.5. (D) Quantification of the relative intensity of p-p70S6K/p70S6K and p-mTOR/mTOR based on western blotting analysis (*n*=3; unpaired, two-tailed Student's *t*-test). (E) The level of Cdc42 activation was measured in both equalized protein concentrations (0.15 µg/µl and 0.61 µg/µl) from control and *Osr2-Cre;Ror2^fl/fl^* (mutant) molars at P3.5 by reading at OD_490_ nm (*n*=3; unpaired, two-tailed Student's *t*-test). (F,G) RNAscope *in situ* hybridization of *Cdc42* (red) and immunostaining of Ki67 (green) on sagittal sections of mandibular molars from wild-type (WT) mice at P3.5. The box in F is shown at higher magnification in G (merged), G′ (*Cdc42*) and G″ (Ki67). The small box in G is shown in the inset at higher magnification to reveal the expression of *Cdc42* and Ki67 in the dental mesenchymal cells. (H) RNAscope *in situ* hybridization of *Cdc42* (red) and immunostaining of Ror2 (green) on sagittal sections of mandibular molars from wild-type mice at P3.5. The small box in H is shown in the inset at higher magnification to reveal the expression of *Cdc42* and Ror2 in the dental mesenchymal cells. (I-R) RNAscope *in situ* hybridization of *Ccne1* (I-L) and *Ccna2* (N-Q) in control and *Osr2-Cre;Ror2^fl/fl^* mandibular molars at P3.5, with quantification of their expression by qPCR (M,R; *n*=3, unpaired, two-tailed Student's *t*-test). Boxes in I, K, N and P are shown at higher magnification in J, L, O and Q, respectively. Arrows indicate positive signals. Dotted lines indicate the border between dental epithelium and dental mesenchyme. Data are mean±s.e.m. Scale bars: 100 µm.

To further probe the molecular mechanism of Ror2-mediated signaling in regulating root development, we analyzed gene expression profiles and performed pathway enrichment analysis in the dental mesenchyme using RNA-seq from *Osr2-Cre;Ror2^fl/fl^* and control mice at P3.5 (Fig. 5A,B; Tables S2, S3). We identified 891 genes that were differentially expressed in *Osr2-Cre;Ror2^fl/fl^* versus control mice (Table S3). These genes segregated into two groups shown in the heat map in Fig. 5A, and interesting candidate genes related to cell proliferation are listed in Table S2.

Pathway enrichment analysis showed that genes related to the regulation of eIF4 and p70S6K signaling were among the most enriched categories and, therefore, were the most likely to contribute to the root size defect in *Ror2* mutant mice (Fig. 5B). The p70S6 kinase (p70S6K, S6K1) is crucially important in protein synthesis, cell growth and cell proliferation (Harada et al., 2001). Therefore, we tested the level of p70S6K in the dental mesenchyme of *Osr2-Cre;Ror2^fl/fl^* and control mice. The results showed that the phosphorylation of p70S6K was significantly decreased in *Osr2-Cre;Ror2^fl/fl^* mice compared with controls (Fig. 5C,D), which is consistent with the signaling pathway analysis. p70S6K is usually regarded as a key factor downstream of the mTOR pathway (Harada et al., 2001). Surprisingly, there was no decrease in the phosphorylation of mTOR in *Ror2* mutant mice (Fig. 5C,D), which indicates that the inactivation of p70S6K may be independent of mTOR signaling in *Osr2-Cre;Ror2^fl/fl^* mouse molars. p70S6K has also been identified as a downstream effector of Cdc42 (Chou and Blenis, 1996), a small Rho-family GTPase. The function of Cdc42 is typically dependent on its switching between an active GTP-bound form and an inactive GDP form. The non-canonical Wnt pathway is important for Cdc42 signaling (Carvalho et al., 2019), and Cdc42 is required for cell cycle progression (Chou et al., 2003). Therefore, we firstly tested the level of GTP-Cdc42 protein in tissue lysates at P3.5 (Fig. 5E). The small G-protein activation assay showed that Cdc42 activation was significantly decreased in *Osr2-Cre;Ror2^fl/fl^* mouse molars in both equalized protein concentrations (0.15 µg/µl and 0.61 µg/µl) (Fig. 5E). We then examined the expression pattern of *Cdc42* in the mouse molar at P3.5. The results showed that there was an overlap in the expression of *Cdc42* and Ki67 in the apical region of the molar root (Fig. 5F-G″), and in the same region there was also overlapping expression between *Cdc42* and Ror2 (Fig. 5H). Furthermore, the dental mesenchymal cells showed a decrease in cell proliferation after treatment with ZCL278 (50 μM), a Cdc42 inhibitor, for 20 h *in vitro* (Fig. S8A-C). These results indicated that Cdc42 may mediate the function of Ror2 in cell proliferation during root development.

Before proceeding with further mechanistic studies, we analyzed gene expression profiles and found that several cell cycle-promoting genes had reduced expression levels in *Osr2-Cre;Ror2^fl/fl^* mice compared with controls (Table S2). Cyclin E1 (Ccne1) and Cyclin A2 (Ccna2) are important regulators of the G1/S transition and S phase of cell cycle progression, respectively (Sonntag et al., 2018). We confirmed that there was a significant reduction in the expression of *Ccne1* and *Ccna2* in the apical mesenchyme *in vivo* at P3.5 by *in situ* hybridization and qPCR (Fig. 5I-R). This indicated that loss of *Ror2* in the dental mesenchyme leads to the downregulation of *Ccne1* and *Ccna2* during root development. Furthermore, we also detected the decreased expression of *Ect2*, which is reported to be related to mitosis, in *Osr2-Cre;Ror2^fl/fl^* molars (Fig. S8D-H). These results were likely associated with the decrease in EdU-labeled cells and PHH3^+^ cells seen in the dental mesenchyme of *Osr2-Cre;Ror2^fl/fl^* molars (Fig. 3I-P). It has been argued that the function of Cdc42 in cell cycle regulation is to promote G1 progression through the induction of *Ccne1* expression (Chou et al., 2003). Therefore, the reduced Cdc42 activation signal observed at P3.5 suggests that Cdc42 may participate in Ror2-induced *Ccne1* expression in tooth root context.

**Fig. 6.**
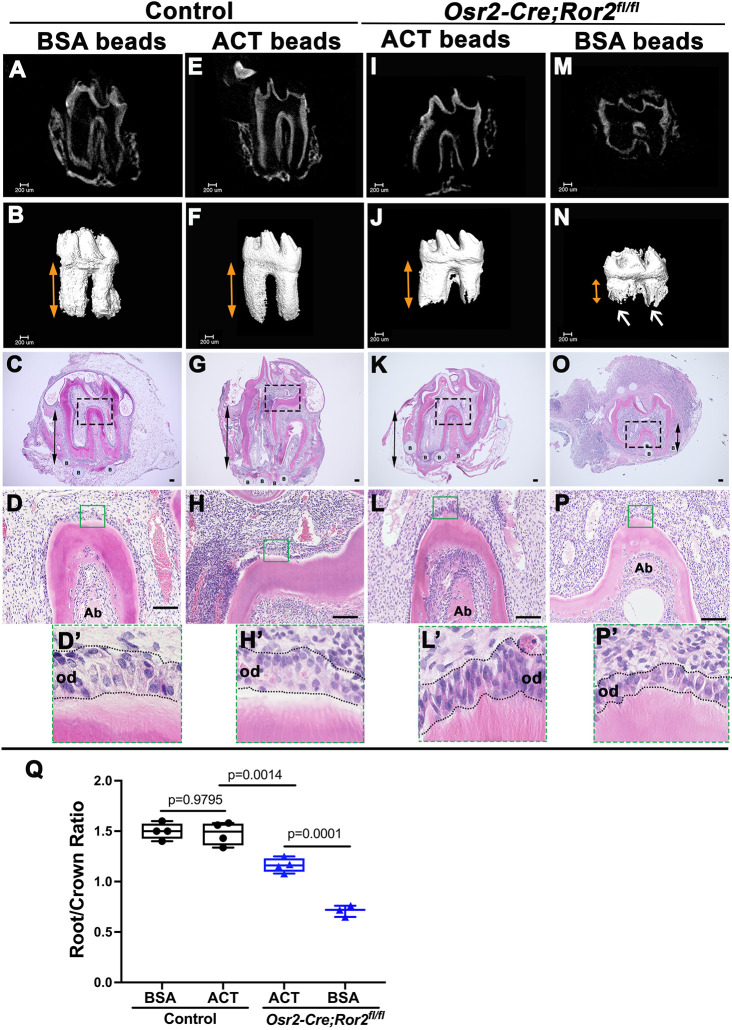
**Cdc42 activator partially rescues root development in *Ror2* mutant mice.** (A-P′) MicroCT images and H&E staining of control (A-H′) and *Osr2-Cre;Ror2^fl/fl^* (I-P′) mandibular molars after 3 weeks of cultivation under kidney capsules with BSA-soaked beads or activator-soaked beads from P4.5. The control explants develop normal root length and root number (*n*=4; A-H). *Osr2-Cre;Ror2^fl/fl^* mandibular molars treated with Cdc42 activator-soaked beads show normal root formation (*n*=4; I-L) but are still shorter than the control molars (A-H). The *Ror2* mutant molars treated with BSA-soaked beads form a shorter root (*n*=3; M-P). Double-headed arrows indicate tooth roots. Boxes in C, G, K and O are shown at higher magnification in D, H, L and P, respectively. Boxes in D, H, L and P show the morphology of odontoblasts (highlighted with dotted lines) in the furcation region at higher magnification in D′, H′, L′ and P′, respectively. (Q) Quantification of the ratio of root to crown in control molars and *Osr2-Cre;Ror2^fl/fl^* molars treated with BSA-soaked beads or activator-soaked beads (two-way ANOVA). The vertical extension of the box and whiskers indicates the dispersion and bias of the data. Data are mean±s.e.m. Ab, alveolar bone; ACT, activator-soaked beads; B, beads; Od, odontoblast. Scale bars: 100 µm.

**Fig. 7.**
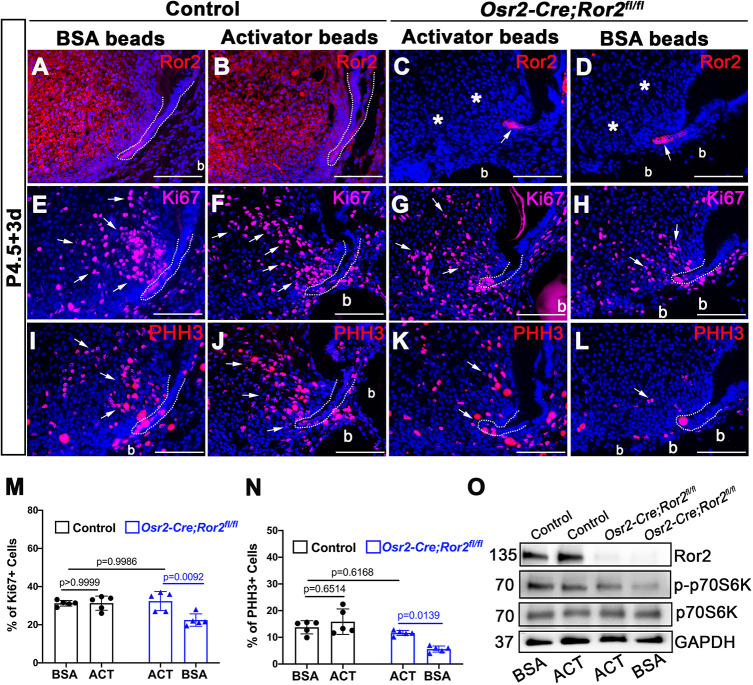
**Cdc42 activator restores cell proliferation in *Ror2* mutant mice.** (A-D) Ror2 expression patterns in control (A,B) and *Osr2-Cre;Ror2^fl/fl^* (C,D) mandibular molars treated with BSA- or Cdc42 activator-soaked beads for 3 days in the kidney capsule from P4.5. Asterisks indicate ablation of Ror2 in the dental mesenchyme. Arrows indicate the presence of Ror2 in dental epithelium. (E-L) Ki67 immunofluorescence (E-H) indicating proliferating cells and PHH3 immunofluorescence (I-L) indicating mitotic cells in the apical region in sagittal sections of mandibular molars treated with BSA- or activator-soaked beads for 3 days in the kidney capsule from P4.5. Arrows indicate positive signals. (M,N) Quantification of Ki67^+^ cells (M) and PHH3^+^ cells (N) in the apical region of control and *Osr2-Cre;Ror2^fl/fl^* molars treated with BSA- or activator-soaked beads for 3 days in the kidney capsule from P4.5 (*n*=5; two-way ANOVA). (O) Western blot of Ror2, p70S6K, p-p70S6K and GAPDH in the dental mesenchyme of control and *Osr2-Cre;Ror2^fl/fl^* molars treated with BSA- or activator-soaked beads for 2 days under the kidney capsule from P4.5. Dotted lines indicate the border between dental epithelium and dental mesenchyme. Data are mean±s.e.m. ACT, activator beads. Scale bars: 100 µm.

Cdc42 is the convergence point between the gene expression profiling and pathway enrichment analyses we conducted. Considering that Cdc42 is involved in non-canonical Wnt signaling and plays an important role in cell cycle regulation (Chou et al., 2003; Olson et al., 1995), we hypothesized that Cdc42 may be an important downstream mediator of Ror2 signaling in regulating root development. To test this hypothesis, we first investigated whether Cdc42 activator could rescue root growth in *Osr2-Cre;Ror2^fl/fl^* mice. We collected tooth germs from control and *Osr2-Cre;Ror2^fl/fl^* mice at P4.5, and transplanted them with bovine serum albumin (BSA)-soaked beads or activator-soaked beads into the kidney capsule. After 3 weeks of cultivation, we harvested the molars for analysis. Based on microCT images and H&E staining, the shortened roots of *Osr2-Cre;Ror2^fl/fl^* molars were partially rescued by Cdc42 activator treatment compared with the controls treated with BSA or activator (*n*=4; Fig. 6A-L′,Q). However, BSA-soaked beads had no significant effect on the root defect in *Osr2-Cre;Ror2^fl/fl^* molars (*n*=3; Fig. 6M-P′,Q). Furthermore, the odontoblasts in the furcation region of *Ror2* mutant molars treated with Cdc42 activator appeared longer than those in the mutant molars treated with BSA beads, and were similar in size to those in the control molars (Fig. 6D,D′,H,H′,L,L′,P,P′). In order to investigate whether the cellular defect was rescued, we examined cell proliferation activity in the apical region at an early stage (Fig. 7A-N). It has been reported that the rescue of gene expression can occur after 2 days of incubation with protein-soaked beads in organ culture (Huang et al., 2010). Therefore, we collected samples 3 days after transplantation under the kidney capsule for analysis in cellular level. We found that the numbers of Ki67^+^ and PHH3^+^ cells were higher in activator-treated *Osr2-Cre;Ror2^fl/fl^* molars than in BSA-treated *Ror2* mutant molars (Fig. 7A-N). However, there was no statistical difference in Ki67/PHH3^+^ cells between activator-treated and BSA-treated controls (Fig. 7E-N). This may explain why there was normal root formation in activator-treated control molars after 3 weeks of cultivation under the kidney capsule. Additionally, p70S6K was also activated with higher phosphorylation in activator-treated *Osr2-Cre;Ror2^fl/fl^* molars compared with BSA-treated *Ror2* mutant molars (Fig. 7O). Furthermore, the expression pattern of *Cdc42* and Ki67 in wild-type tooth germ was similar to that at P7.5 *in vivo* (Fig. S8I-L). Therefore, we concluded that activation of Cdc42 contributes to the restoration of cell proliferation in the apical region and root formation in *Osr2-Cre;Ror2^fl/fl^* molars. These results highlight the significance of Cdc42 as an effective mediator of Ror2 in regulating cell proliferation during root morphogenesis.

## DISCUSSION

Previous studies have reported that several signaling pathways, including Bmp, Tgfβ, Wnt, Fgf and Shh, are important in regulating root formation. However, the function of non-canonical Wnt signaling in root development has remained unknown. Ror2 crucially regulates the morphogenesis of a variety of organs during embryonic development (Minami et al., 2010). Here, we have provided evidence that Ror2 plays a crucial role in regulating cell proliferation in the apical region of the mouse molar, and consequently in regulating root size during development. Additionally, we identified Cdc42 as a potential downstream mediator of Ror2 signaling in root formation associated with mesenchymal cell proliferation during postnatal tooth morphogenesis.

### Mesenchymal Ror2 mediates epithelial-mesenchymal interactions during root development

Sequential reciprocal epithelial-mesenchymal interactions are crucial for virtually every aspect of tooth morphogenesis and development (Balic and Thesleff, 2015). Both epithelial and mesenchymal cells contribute to the formation of the tooth root (Li et al., 2017). Previous studies have shown that HERS is important for the induction of odontoblast differentiation (Kim et al., 2013) and may be responsible for determining tooth root size, shape and number (Li et al., 2017). On the molecular level, HERS secretes Shh under the control of Tgfβ/Bmp/Smad, and in so doing regulates mesenchymal *Nfic* expression to control root elongation (Huang et al., 2010), suggesting that HERS acts as a signaling center that mediates epithelial-mesenchymal interactions. Some signaling molecules in the dental mesenchyme are also essential for HERS formation, as well as for root formation. For example, conditional knockout of *β-catenin* or *Tgfbr2* in odontoblasts disrupts root odontoblast differentiation and HERS formation, which leads to molar root defects (Kim et al., 2013; Wang et al., 2013). In this study, we provide support for the notion that *Ror2* is not required in the dental epithelium during root formation, but mesenchyme-specific deletion of *Ror2* results in shortened roots in *Osr2-Cre;Ror2^fl/fl^* mice, suggesting that Ror2-mediated signaling is specifically required in the dental mesenchyme during root development.

Soon after birth, *Osr2-Cre;Ror2^fl/fl^* mice exhibit decreased mesenchymal proliferation, followed by HERS malformation with a less distinct invagination into the mesenchyme and disrupted odontoblast differentiation. A previous study has shown that spatial regulation of mesenchymal proliferation is required for root patterning, and that this process may involve the mesenchyme forming a physical structure that affects the epithelial invagination of HERS (Sohn et al., 2014). In light of this, we suggest that the HERS malformation seen in *Osr2-Cre;Ror2^fl/fl^* mice may be caused at least in part by the physical effect of the dental mesenchyme resulting from its altered proliferation. Other factors secreted from the dental mesenchyme may also have affected HERS formation through tissue-tissue interactions. Although it is still unknown how HERS formation is specifically affected in *Ror2* mutant mice, the evidence suggests that mesenchymal Ror2 is important for modulating epithelial-mesenchymal interactions during root development. We found different morphological changes to odontoblasts in the elongation and furcation regions of the root. These differences may result from the site-specific regulation of *Ror2* or from the distinct mechanisms underlying root elongation and furcation formation. For example, *Osx^Col^* and *Osx^OC^* mice have also been reported to form short roots with extremely thin inter-radicular dentin (Kim et al., 2015). In *Osr2-Cre;Ror2^fl/fl^* mice, the disrupted differentiation of odontoblasts in the elongation region may be related to decreased mesenchymal proliferation causing fewer cells to withdraw from the cell cycle for differentiation.

### Ror2-Cdc42 signaling regulates root development

Although Ror2 can mediate the canonical Wnt pathway (Billiard et al., 2005; Roarty et al., 2017), we show that loss of *Ror2* in the dental mesenchyme has no effect on β-catenin-dependent transcriptional activity when tooth root development initiates. Therefore, we have focused on Ror2-mediated non-canonical Wnt signaling in our mechanistic study, specifically the function of Cdc42 activated by Ror2 in root formation.

*CDC42* mutations result in a spectrum of human diseases with various clinical phenotypes, such as developmental delay, growth retardation, facial dysmorphism and more (Szczawinska-Poplonyk et al., 2020). In mouse models, epithelial Cdc42 deletion induces enamel defects and dental cystogenesis, suggesting it may play a role in tooth development (Melendez et al., 2013). Cdc42 is essential in various cellular processes. For example, non-canonical Wnt-dependent Cdc42 activity is required for tissue repair after neural demyelination injury (Chavali et al., 2018). The Ror2-Cdc42 signaling axis has also been reported to mediate Wnt5a signaling transduction to regulate junctional mechano-coupling between endothelial cells (Carvalho et al., 2019). Interestingly, deletion of *Cdc42* in the neural crest (NC) results in multiple defects in NC derivatives because of reduced mitotic activity (Fuchs et al., 2009). In our study, deletion of *Ror2* in the dental mesenchyme results in decreased Cdc42 activity and cell proliferation in *Osr2-Cre;Ror2^fl/fl^* mice. Cdc42 activator can restore cell proliferation in the apical region of mouse molars and partially rescue the shortened root phenotype in *Ror2* mutant mice. These findings imply that Cdc42 is a key downstream mediator of Ror2 signaling during root development.

Ror2 regulates Cdc42 activation through Phosphoinositide 3 kinase, as has been described in *Xenopus* gastrulation (Schambony and Wedlich, 2007). It has also been reported that Cdc42 acts downstream of the Ca^2+^ signaling pathway triggered by Wnt5a binding to the Ror2-Frizzled receptor complex (Jin et al., 2005; Li et al., 2009; Zhan et al., 2016). In our study, pathway enrichment analysis has shown that Ca^2+^ signaling is downregulated in *Ror2* mutant mice. We also detect the decreased expression of calcineurin A (a downstream mediator of Ca^2+^ signaling) and transcription factor *Nfatc4* in the apical region of mouse molars, confirming the downregulation of Ca^2+^ signaling in *Osr2-Cre;Ror2^fl/fl^* mice. It remains to be investigated whether altered Ca^2+^ signaling caused by loss of *Ror2* in the dental mesenchyme plays an important role in modulating the activity of Cdc42 to regulate root size during tooth morphogenesis.

Consistent with the inhibition of Cdc42 activity in *Osr2-Cre;Ror2^fl/fl^* mice, the phosphorylation of p70S6K, an effector of Cdc42, is significantly decreased in the dental mesenchyme. In accordance with this finding, p70S6K activity has been increased after Cdc42 activator treatment. The phosphorylation of p70S6K is beneficial for protein synthesis required both to enter the cell cycle (G1 phase) from G0 and to proceed to S phase (Pearson and Thomas, 1995). Therefore, the cell proliferation in the apical region is restored after Cdcd42/p70S6K is activated in *Ror2* mutant molars. Although it is well documented that p70S6K is a downstream target of the mTOR pathway, this is not the case in *Ror2* mutant mice during tooth root development. *In vitro* study has shown that Cdc42 can act through both mTOR-dependent and -independent pathways to regulate p70S6K in HEK293 cells (Fang et al., 2003). Therefore, we suggest that the lack of the upstream signal from Ror2 results in dephosphorylation and inactivation of p70S6K in the dental mesenchyme, in which it may be independent of the mTOR pathway but requires the involvement of Cdc42. It has also been reported that Cdc42 has multiple functions in regulating the cell cycle (Chou et al., 2003; Olson et al., 1995); for example, it can regulate mitosis via modulating chromosome alignment (Yasuda et al., 2006). Therefore, the Cdc42-p70S6K axis may not be the only pathway associated with Cdc42 in regulating cell proliferation in the dental mesenchyme. We cannot exclude other ways in which Cdc42 functions in cell cycle regulation. Collectively, our findings indicate that Cdc42 is an important downstream target of Ror2 signaling that effectively modulates cell proliferation during root development.

In summary, our study demonstrates that the Ror2-mediated signaling pathway in the dental mesenchyme plays a crucial role in regulating molar root development via Cdc42 activation. However, loss of *Ror2* in the dental epithelium does not affect root development. Our findings suggest that Ror2 signaling is tissue specific during root morphogenesis. Importantly, the shortened root size caused by loss of *Ror2* in the dental mesenchyme is similar to the dental problems seen in some individuals with RRS who have mutations in *ROR2* (Jain et al., 2017). The sequences of mouse and human *Ror2*/*ROR2* are almost identical (Stricker et al., 2017). Therefore, a better understanding of the underlying mechanism involved in mouse tooth root development could shed light on the dental defects seen in individuals, and suggest potential avenues for future root regeneration.

## MATERIALS AND METHODS

### Generation of transgenic mouse lines

*Osr2-Cre* mice were a gift from Rulang Jiang (Cincinnati Children's Hospital, OH, USA) (Lan et al., 2007)*. K14-rtTA* (007678; Xie et al., 1999), *Teto-Cre* (006234; Perl et al., 2002), *Ror2^fl/fl^* (018354; Ho et al., 2012), and *ROSA26^loxP-STOP-loxP-tdTomato^* (*tdTomato* conditional reporter, 007905; Madisen et al., 2010) mouse lines were purchased from The Jackson Laboratory. *Osr2-Cre;Ror2^fl/+^* male mice were crossed with *Ror2^fl/fl^* female mice to generate *Osr2-Cre;Ror2^fl/fl^* mice. *K14-rtTA;Teto-Cre;Ror2^fl/fl^* male mice were crossed with *K14-rtTA;Ror2^fl/fl^* female mice to generate *K14-rtTA;Teto-Cre;Ror2^fl/fl^* mice. A doxycycline rodent diet was administered every day from E14.5. All animal studies were approved by the Institutional Animal Care and Use Committee (IACUC) at the University of Southern California (USC).

### Kidney capsule transplantation

Kidney capsule transplantation was performed as described previously (Oka et al., 2007). After genotyping, the tooth germs of the mandibular first molars were collected from P4.5 *Ror2^fl/fl^* control or *Osr2-Cre;Ror2^fl/fl^* mice. Each tooth germ was surgically transplanted under the kidney capsule of a host mouse. The explants were harvested after 21 days. For the rescue experiment, affi-Gel blue agarose beads (Bio-Rad) were soaked in BSA (100 μg/ml) or the Cdc42 activator (50 μg/ml; Cytoskeleton, Inc.) overnight at 4°C before use. After that, BSA and activator beads were transplanted into the kidney capsule with the tooth germ collected from *Ror2^fl/fl^* control or *Osr2-Cre;Ror2^fl/fl^* mouse mandibles at P4.5. The explants were collected after 2, 3 or 21 days.

### MicroCT analysis

MicroCT analysis was performed using a SCANCO µCT50 device at the USC Molecular Imaging Center. The microCT images were acquired with the X-ray source at 70kVp and 114 µA. The data were collected at a resolution of 16.7 µm. Three-dimensional reconstruction was performed using AVIZO 7.1 (Visualization Sciences Group).

### Histological analysis

Samples were dissected and fixed in 4% paraformaldehyde (PFA) overnight at 4°C and decalcified in 10% EDTA (pH 7.5) at 4°C for 1-3 weeks depending on the age of samples. Then samples were processed into paraffin-embedded serial sections at 5 µm using a microtome (Leica). For general morphology, deparaffinized sections were stained with H&E using standard procedures.

For cryosections, decalcified samples were dehydrated in 15% and 60% sucrose/DEPC-treated PBS solution overnight at 4°C. Samples were then embedded in optimal cutting temperature compound (Tissue-Tek). Embedded samples were then cryosectioned at 8 µm using a cryostat (Leica CM1850).

### Immunostaining

Immunostaining was carried out using primary antibodies against Ror2 (1:100, Cell Signaling Technology, 886395), Ki67 (1:100, Abcam, ab15580), K14 (1:200, Abcam, ab181595), amelogenin X (AMELX, 1:200, Abcam, ab153915), phospho-ATF2 (1:100, Abcam, ab32019), phospho-c-Jun (1:100, Abcam, ab30620), phospho-histone H3 (1:100, MilliporeSigma, 06-570) and active β-catenin (1:100, Cell Signaling Technology, 19807s). Alexa Fluor 647, Alexa Fluor 568, Alexa Fluor 488 (1:200, Invitrogen) and Alexa Fluor 488 Tyramide SuperBoost kit (Invitrogen, B40922) were used for detection. Sections were counterstained with DAPI.

### *In situ* hybridization

*In situ* hybridization was carried out following standard procedure using an RNAscope 2.5 HD Reagent Kit-RED (ACDBio, 322350) and RNAscope Multiplex Fluorescent Reagent kit V2 Assay (ACDBio, 323100-USM). All probes from mouse cDNA clones were purchased from ACDBio, including RNAscope Probe-Mm-*Dspp* (448301), -*Cdc42* (473041), -*Ccne1* (503801), -*Ccna2-C3* (442661-C3), -*Ect2* (502121) and -*Axin2* (400331).

### Western blotting

Dental pulp of the mandibular first molars was dissected from *Ror2^fl/fl^* control mice and *Osr2-Cre;Ror2^fl/fl^* mice at P3.5. For kidney capsule transplantation, dental pulp of transplanted molars was dissected from the kidney capsule 2 days post-surgery. For western blotting, tissues were lysed in lysis buffer [50 mMTris-HCl (pH 7.5), 150 mM NaCl, 2 mM EDTA, 0.1% NP-40, 10% glycerol and protease inhibitor cocktail] and protein fractions were isolated. Proteins were separated on 4-15% protein gels (Bio-Rad) and transferred to a 0.2 µm PVDF membrane. Membranes were blocked in 5% BSA for 1 h at room temperature and then incubated with primary antibody overnight at 4°C, followed by 1 h incubation with horseradish peroxidase-conjugated anti-mouse IgG or anti-rabbit IgG secondary antibody. Immunoreactive protein was detected using SuperSignal West Femto Maximum Sensitivity Substrate (Thermo Fisher Scientific, A10044).

The primary antibodies used for western blotting were as follows: anti-GAPDH (1:1000, Cell Signaling Technology, 5174s); anti-Ror2 (1:1000, Cell Signaling Technology, 886395); anti-Cdc42 (1:1000, Abcam, ab64533); anti-phospho-p70S6 kinase (1:1000, Cell Signaling Technology, 9205S); anti-p70S6K (1:1000, Cell Signaling Technology, 2708); anti-mTOR (1:1000, Cell Signaling Technology, 2972s); and anti-phospho-mTOR (1:1000, Invitrogen, 44-1125G).

### Cdc42/Rac1 G-LISA activation assay

Dental pulp of the mandibular first molars was dissected from *Ror2^fl/fl^* control mice and *Osr2-Cre;Ror2^fl/fl^* mice at P3.5. Then tissues were lysed in lysis buffer including protease inhibitor cocktail (Cytoskeleton, PIC02) and protein fractions were isolated. According to the recommended protocol, GTP-loaded Cdc42 or Rac1 protein in tissue lysates was detected using Cdc42 or Rac1 G-LISA assay, respectively (Cytoskeleton, BK127/BK128). The level of activation was measured by reading at OD_490_ nm.

### TUNEL assays

Apoptotic cells in mouse molars were detected using the Click-iT Plus TUNEL assay (Thermo Fisher Scientific, C10617) according to the recommended protocol.

### EdU incorporation and staining

To test cell proliferation, the *Ror2^fl/fl^* control and *Osr2-Cre;Ror2^fl/fl^* mice were treated with a 2 h pulse of EdU (25 ug/g body weight) administered intraperitoneally at P3.5. For cell differentiation analysis, EdU was injected intraperitoneally at P0.5 and samples were collected at P2.5. Specimens were fixed, decalcified and sectioned as described for histological analysis. EdU was detected using a Click-iT Plus EdU Cell Proliferation kit (Thermo Fisher Scientific, C10637). Sections were counterstained with DAPI.

### RNA-seq analysis

Dental pulp of the mandibular first molar was dissected out from *Ror2^fl/fl^* control mice and *Osr2-Cre;Ror2^fl/fl^* mice at P3.5. RNA was then extracted using an RNeasy Micro kit (Qiagen). cDNA library preparation and sequencing were performed at the University of California, Los Angeles Technology Center for Genomics and Bioinformatics. A total of 200 million single-end reads with 75 cycles were performed on Illumina Hiseq 4000 equipment for three pairs of samples. High-quality reads were aligned using TopHat 2 with the mm10 genome. Data were quantified by counting the number of reads over exons and normalized using reads per kilobase of transcript per million mapped reads. Differential expression was estimated by selecting transcripts that changed with a significance of *P*<0.05.

### Cell culture *in vitro* assay

The apical half of the dental pulp mesenchyme of the mandibular first molar was dissected out from wild-type mice at P3.5. The tissue was minced, centrifuged and resuspended in α-minimum essential medium (MEM) with 20% fetal bovine serum (FBS), 2 mM L-glutamine, 55 μM β-mercaptoethanol, 100 U/ml penicillin and 100 μg/ml streptomycin (Life Science Technologies). After 5 days of cultivation, cells were digested and obtained by passing through a 40 um strainer (Corning). The cell suspension was seeded at 3.5×10^4^/well in 12-well plate culture (Corning) and incubated for 48 h at 37°C with 5% O_2_ and 5% CO_2_. The medium was then changed using fresh α-MEM without FBS for 10 h. Serum-starved mesenchymal cells were then incubated for another 20 h with DMSO as control or 50 μM ZCL278 (Cdc42 inhibitor; Tocris, 4794). For detection of PHH3, cells were washed with PBS before fixation with 4% PFA in PBS at room temperature for 20 min. Antibody to PHH3 was used at 1:100 dilution. Alexa Fluor 647 was used for detection. Samples were counterstained with DAPI.

### qPCR analysis

RNA was isolated from the dental pulp of the mandibular first molars from *Ror2^fl/fl^* control mice and *Osr2-Cre;Ror2^fl/fl^* mice at P3.5 using an RNeasy Micro kit (Qiagen). An iScript cDNA Synthesis kit (Bio-Rad) was used for cDNA synthesis. qPCR was then performed using an iCycler (Bio-Rad) with gene-specific primers, SYBR Green and an SsoFast EvaGreen Supermix kit (Bio-Rad). Values were normalized to *Gapdh* using the 2^−ΔΔCt^ method. The PCR primers are listed in Table S1.

### Statistical analysis

Data are presented as mean±s.e.m. and were analyzed using Prism 8 (GraphPad Software). *n* represents the number of control or mutant mice, or the number of independent experiments performed. We used *n*≥3 for all experiments unless otherwise noted. For each independent experiment, including qPCR and western blotting, at least three technical replicates were analyzed. For immunostaining and *in situ* hybridization, we used samples from at least three individual mice, and representative images from one of three sections of one sample are shown. For *in vivo* studies, statistical comparisons were performed using unpaired Student's *t*-test with two-tailed calculations. For *ex vivo* studies, statistical significance was determined using two-way ANOVA, followed by Tukey's multiple comparison tests. *P*<0.05 was considered statistically significant for all analyses.

## Supplementary Material

Supplementary information

Reviewer comments
