# Supplementary material for: Ror2-mediated non-canonical Wnt signaling regulates Cdc42 and cell proliferation during tooth root development
Source: Development. 2021 Jan 21;148(2):dev196360. doi: 10.1242/dev.196360 (PMC7847279; doi:10.1242/dev.196360)
Supplement: Supplementary information [file develop-148-196360-s1.pdf]

SUPPLEMENTARY INFORMATION

Supplementary Figures

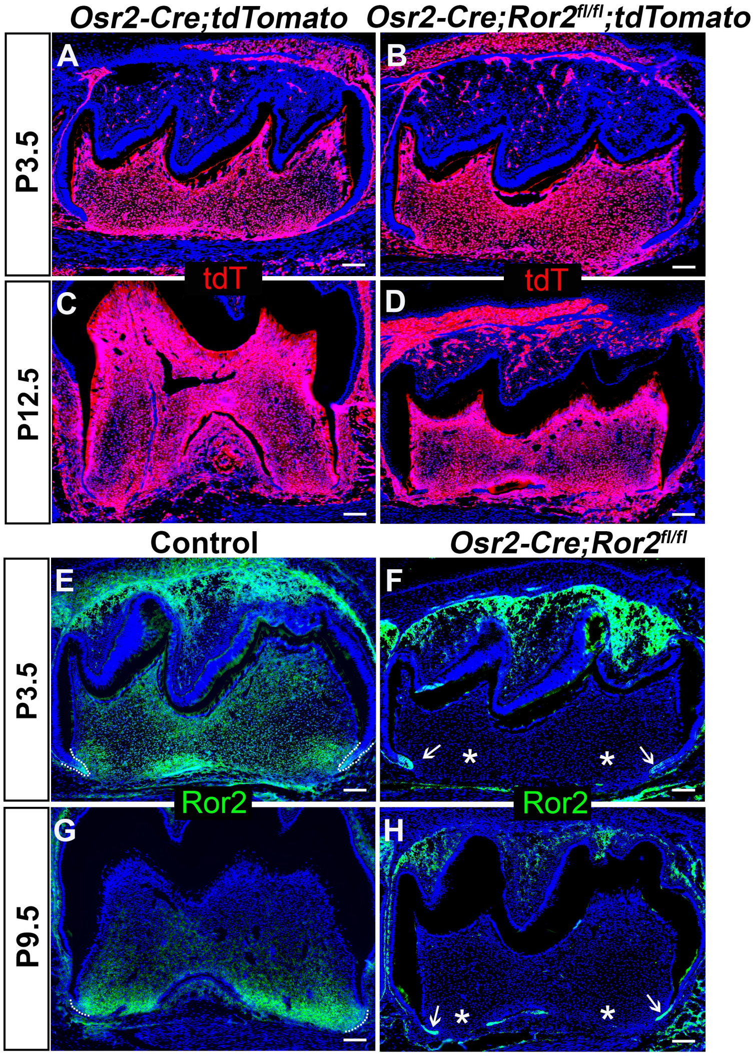

Figure S1. Ror2 is efficiently deleted in the dental mesenchyme in *Osr2-Cre;Ror2* $^{fl/fl}$ mice. (A-D) Visualization of tdTomato of sagittal sections of molars from *Osr2-Cre;tdTomato* and *Osr2-Cre;Ror2* $^{fl/fl}$*;tdTomato* mice at P3.5 (A, B) and P12.5 (C, D). (E-H) Ror2 expression patterns in control and *Osr2-Cre;Ror2* $^{fl/fl}$ mandibular molars at P3.5 (E, F) and P9.5 (G, H). Asterisks indicate ablation of Ror2 in the dental mesenchyme. Arrows indicate the presence of Ror2 in dental epithelium. tdT, tdTomato. Scale bars:100 µm.

Development • Supplementary information

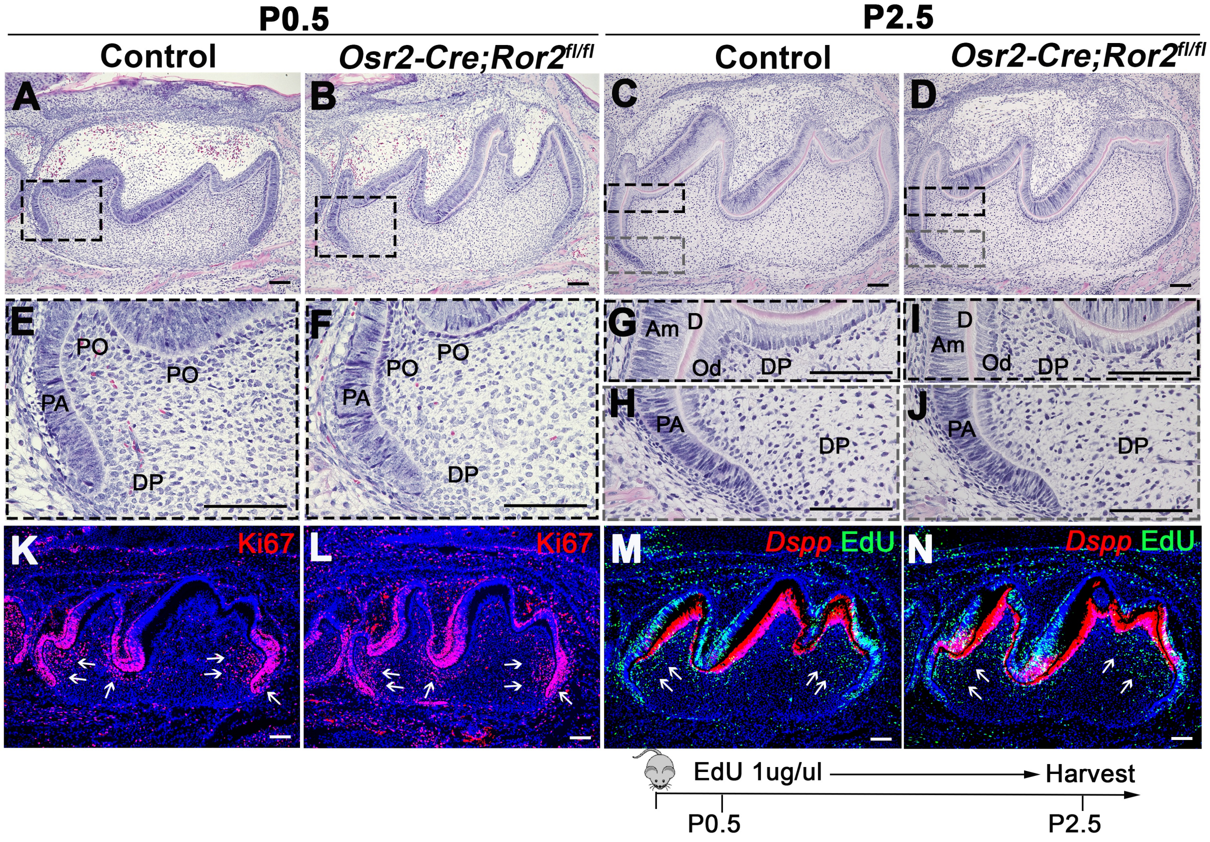

Figure S2. Loss of *Ror2* in the dental mesenchyme does not affect crown formation.

(A-J) Histological analysis of control and *Osr2-Cre;Ror2* $^{fl/fl}$ mandibular molars at P0.5 (A, B, E, F) and P2.5 (C, D, G, H, I, J). Boxes in A and B are enlarged in E and F, respectively. Boxes in C are enlarged in G and H, and boxes in D are enlarged in I and J. DP, dental pulp; PA, pre-ameloblast; PO, pre-odontoblast; Am, ameloblast; Od, odontoblast; D, dentin. (K, L) Ki67 immunofluorescence (red) indicating proliferating cells in sagittal sections of mandibular molars in control (K) and *Osr2-Cre;Ror2* $^{fl/fl}$ (L) mandibular molars at P0.5. Arrows indicate positive signals. (M, N) RNAscope *in situ* hybridization of *Dspp* (red) and EdU assay (green) in control (M) and *Osr2-Cre;Ror2* $^{fl/fl}$ (N) mandibular molars at P2.5, two days after EdU injection at P0.5. Arrows indicate EdU positive signals. Schematic at the bottom indicates injection protocol. Scale bars:100 μm.

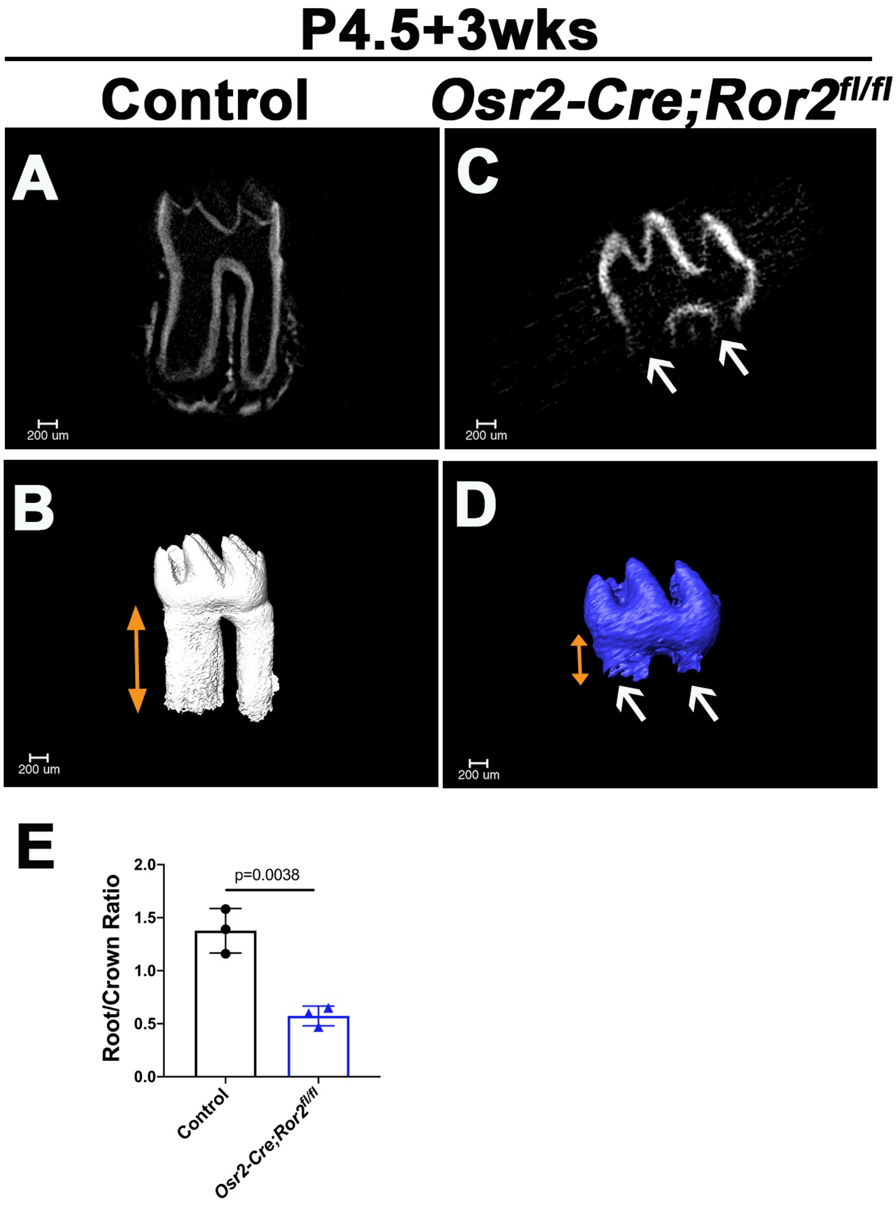

Figure S3. Loss of *Ror2* leads to shortened roots in *Osr2-Cre;Ror2* $^{fl/fl}$ molars in kidney capsule transplantation. MicroCT images of control (A, B) and *Osr2-Cre;Ror2* $^{fl/fl}$ (C, D) mandibular molars after 3 weeks of cultivation under kidney capsules since P4.5. (E) Quantification of the ratio of root to crown in control molars and *Osr2-Cre;Ror2* $^{fl/fl}$ molars cultivated under kidney capsule. Arrows indicate the apical region of the roots and double-headed arrows indicate tooth roots.

Development • Supplementary information

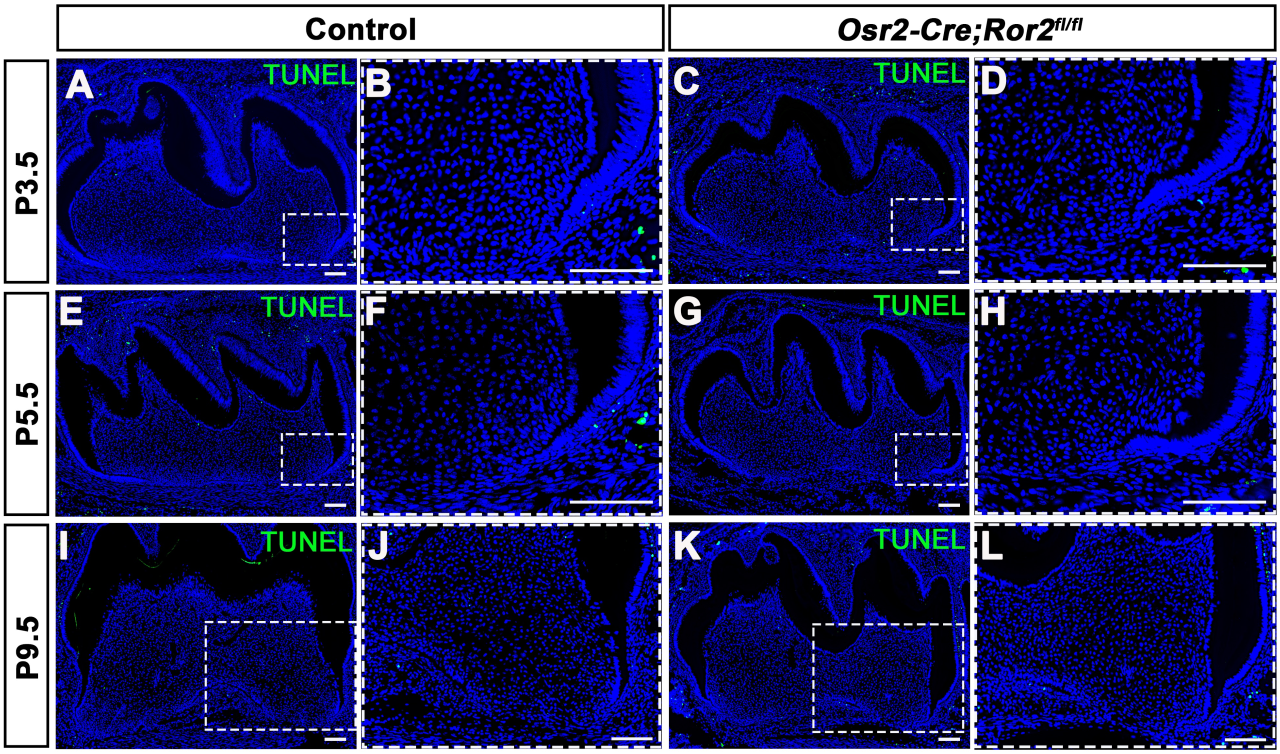

Figure S4. Cell apoptosis is unchanged in the apical region of *Osr2-Cre;Ror2* $^{fl/fl}$ mandibular molars. (A-L) TUNEL assay of sagittal sections of mandibular molars from control and *Osr2-Cre;Ror2* $^{fl/fl}$ mice at P3.5 (A-D), P5.5 (E-H) and P9.5 (I-L). Boxes in A, C, E, G, I and K are enlarged in B, D, F, H, J and L, respectively. Scale bars:100 µm.

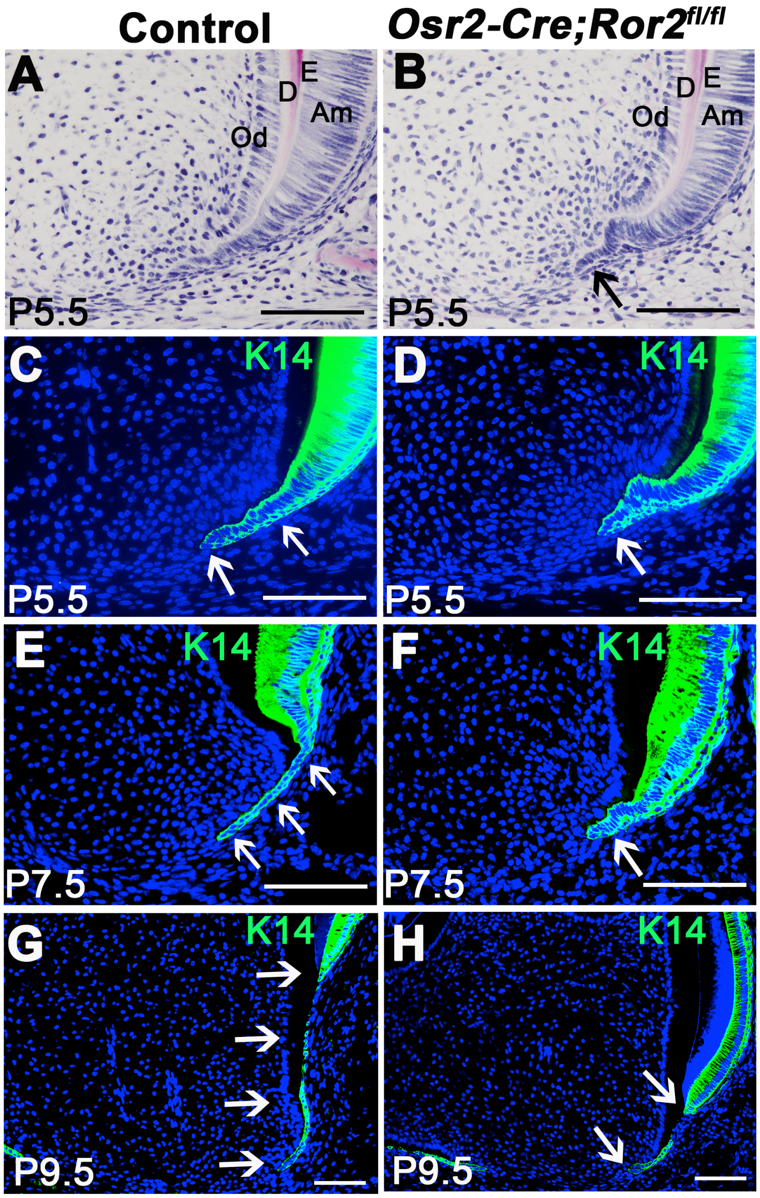

Figure S5. Epithelial diaphragm is compromised through tissue-tissue interaction after loss of *Ror2* in dental mesenchyme. (A, B) H&E staining of control (A) and *Osr2-Cre;Ror2* $^{fl/fl}$ (B) mandibular molars at P5.5. Od, odontoblast; D, dentin; E, enamel; Am, ameloblast. Arrows indicate HERS. (C-H) K14 immunofluorescence (green) indicating the dental epithelium in sagittal sections of control and *Osr2-Cre;Ror2* $^{fl/fl}$ mandibular molars at P5.5 (C, D), P7.5 (E, F) and P9.5 (G, H). Arrows indicate HERS. Scale bars:100 µm.

Development • Supplementary information

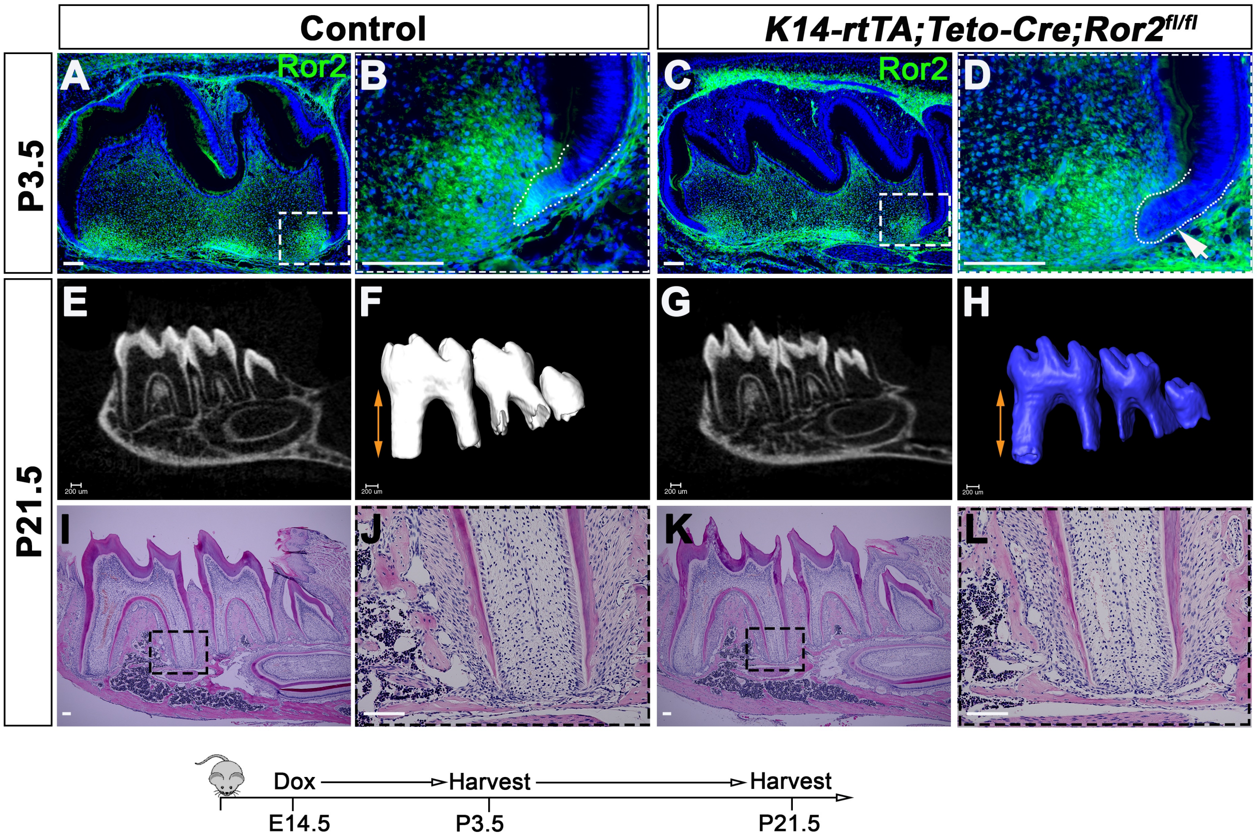

Figure S6. Loss of *Ror2* in the dental epithelium has no effect on root development.

(A-D) Ror2 is effectively deleted in the dental epithelium in *K14-rtTA;Teto-Cre;Ror2* $^{fl/fl}$ mice (C, D), compared to control mice (A, B) at P3.5. Dotted lines indicate the border between dental epithelium and dental mesenchyme. The arrow indicates the lack of positive signal in the dental epithelium. Scale bars:100 µm. (E-H) MicroCT images of control (E, F) and *K14-rtTA;Teto-Cre;Ror2* $^{fl/fl}$ (G, H) mandibular molars at P21.5. Double-headed arrows indicate tooth roots. (I-L) Histological analysis of control (I, J) and *K14-rtTA;Teto-Cre;Ror2* $^{fl/fl}$ (K, L) mandibular molars at P21.5. Boxes in A, C, I and K are enlarged in B, D, J and L, respectively. Schematic at the bottom indicates induction protocol. Scale bars:100 µm.

Development • Supplementary information

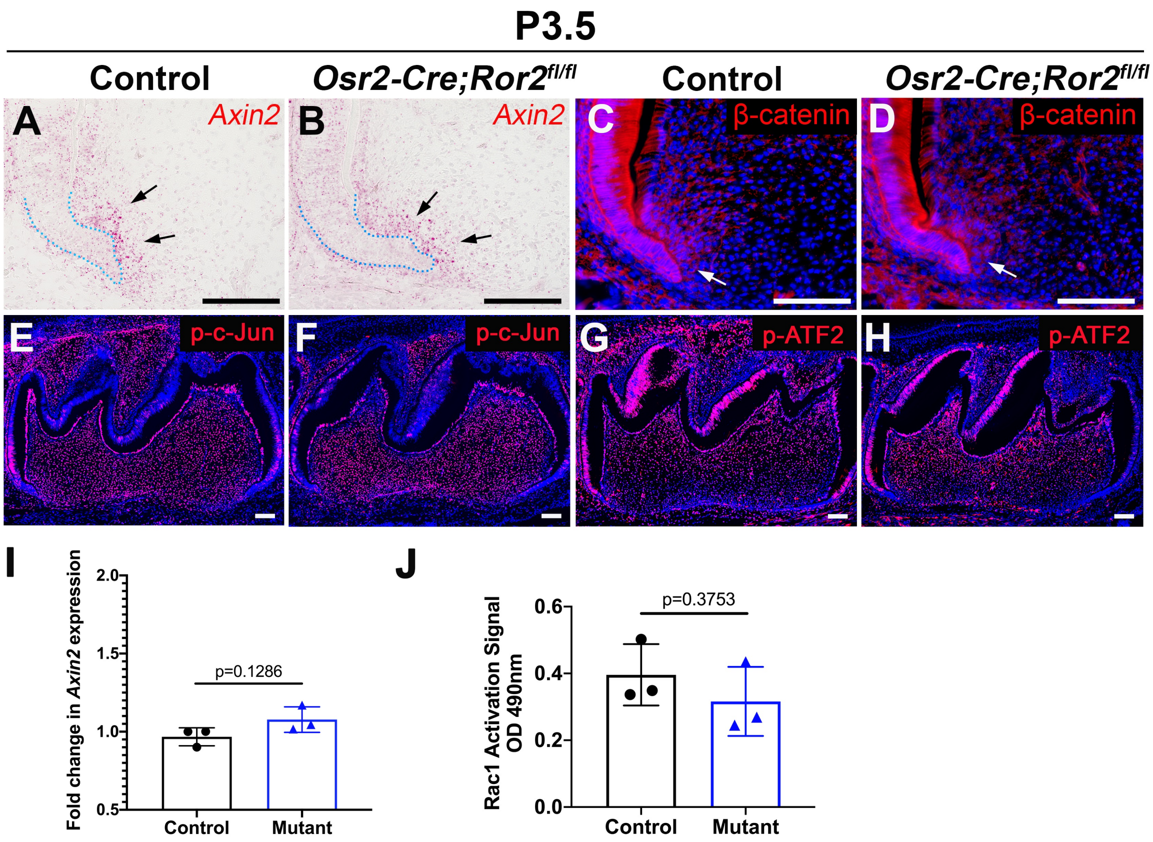

Figure S7. Canonical Wnt pathway and c-Jun/ATF2 activation are unaffected in *Osr2-Cre;Ror2* $^{fl/fl}$ mandibular molars. (A-D**)** RNAscope *in situ* hybridization of *Axin2* (A, B) and immunostaining of active β-catenin (C, D) in the apical region of molars from control and *Osr2-Cre;Ror2* $^{fl/fl}$ mice at P3.5. Dotted lines indicate the border between dental epithelium and dental mesenchyme. Arrows indicate positive signals. (E-H) Phospho-c-Jun (p-c-Jun; E, F) and phospho-ATF2 (p-ATF2; G, H) immunostaining of sagittal sections of molars from control and *Osr2-Cre;Ror2* $^{fl/fl}$ mice at P3.5. Scale bars:100 µm. (I) Fold change of *Axin2* expression in the dental mesenchyme from control and *Osr2-Cre;Ror2* $^{fl/fl}$ (mutant) mandibular molars at P3.5. (J) The level of Rac1 activation was measured in tissue lysates from the dental mesenchyme in control and *Osr2-Cre;Ror2* $^{fl/fl}$ (mutant) molars at P3.5 by reading at OD490nm.

Development • Supplementary information

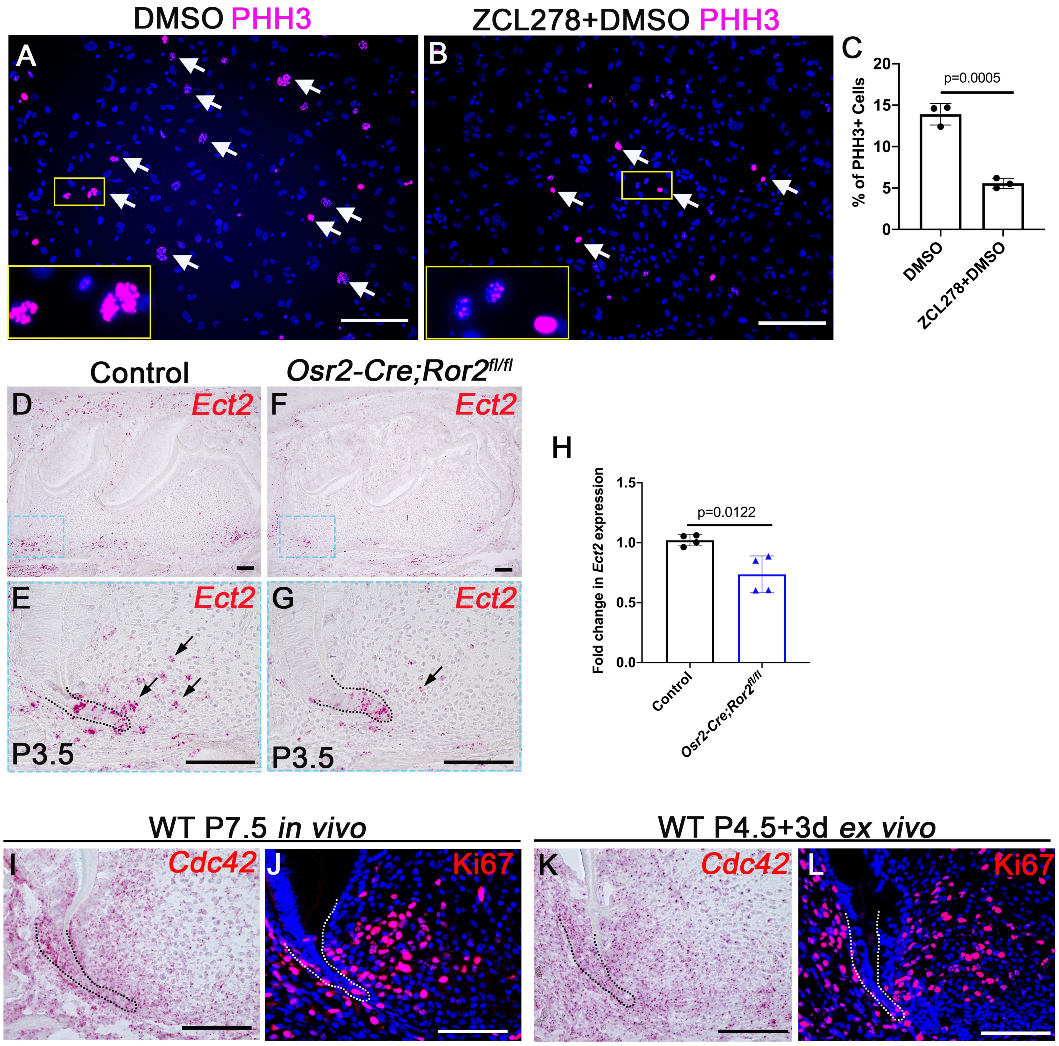

Figure S8. Putative downstream targets after loss of *Ror2* in dental mesenchyme.

(A-C) Immunofluorescence light microscopy of PHH3 in mesenchymal cells treated with DMSO (control; A) or Cdc42 inhibitor ZCL278 (B) and quantification of PHH3-positive cells (C). The small box in A/B is shown in the inset at higher magnification. Arrows indicate positive signals. (D-H) RNAscope *in situ* hybridization of *Ect2* in control (D, E) and *Osr2-Cre;Ror2* $^{fl/fl}$ (F, G) mandibular molars at P3.5 with quantification of fold change in gene expression by qPCR (H). Boxes in D and F are shown at higher magnification in E and G, respectively. Arrows indicate positive signals. (I-L) RNAscope *in situ* hybridization of *Cdc42* and immunostaining of Ki67 in wild-type molars at P7.5 *in vivo* (I, J) and after 3 days of cultivation under kidney capsule since P4.5 (K, L). Dotted lines indicate the border between dental epithelium and dental mesenchyme. Scale bars:100 µm.

Development • Supplementary information

Supplementary Tables

Table S1. List of PCR primers.

Gene	Forward sequence	Reverse sequence	Application
Gapdh	AGGTCGGTGTGAACGGATTTG	GGGGTCGTTGATGGCAACA	qRT-PCR
Ccne1	TGCACCAGTTTGCTTATGTT	CCGTGTCGTTGACATAGG	qRT-PCR
Ccna2	GGCTGACACTCTTTCCG	CTGGTAGCAAGAATTAGAGCAT	qRT-PCR
Axin2	AACCTATGCCCGTTTCCTCTA	GAGTGTAAAGACTTGGTCCACC	qRT-PCR
Ect2	AACTTGTGCTTGGCGTCTAC	CTCCCCTTTGTGCACAGTTG	qRT-PCR

Table S2. The 15 selected candidate genes generated from RNA-sequencing analysis

Gene Symbol	Gene Name	Total counts	Fold change
Cnbd2	*Cyclic nucleotide-binding domain-containing protein 2*	2.73E+01	3.05
Ccne1	*Cyclin E1*	1.37E+01	-1.12
Ect2	*Epithelial cell transforming gene 2*	2.48E+01	-1.53
Ccnc	*Cyclin C*	7.08E+01	-1.61
Taf13	*Transcription initiation factor TF IID subunit 13*	8.17E+01	-1.65
Dock11	*Dedicator of cytokinesis protein 11*	5.61E+01	-1.71
Ccna2	*Cyclin A2*	6.84E+01	-1.74
Ccng1	*Cyclin G1*	3.67E+02	-1.75
Cbx3	*Chromobox Protein homolog 3*	4.70E+02	-1.94
Fgd3	*FYVE, RhoGEF and PH domain containing 3*	3.93E+01	-1.94
Vcan	*Versican*	2.34E+03	-2.01
Fgfr3	*Fibroblast growth factor receptor 3*	2.25E+02	-2.11
Rps6	*40S ribosomal protein S6*	2.56E+02	-2.21
Pdgfd	*Platelet-derived growth factor D*	1.53E+02	-2.51
Fgf13	*Fibroblast growth factor 13*	6.38E+01	-2.57

Table S3. All 891 differentially expressed genes generated from RNA-sequencing analysis

Click here to Download Table S3

Development • Supplementary information
