# Supplementary material for: Ror2-mediated non-canonical Wnt signaling regulates Cdc42 and cell proliferation during tooth root development
Source: Development. 2021 Jan 21;148(2):dev196360. doi: 10.1242/dev.196360 (PMC7847279; doi:10.1242/dev.196360)
Supplement: Reviewer comments [file develop-148-196360_review_history.pdf]

# Ror2-mediated non-canonical Wnt signaling regulates Cdc42 and cell proliferation during tooth root development

Yuanyuan Ma, Junjun Jing, Jifan Feng, Yuan Yuan, Quan Wen, Xia Han, Jinzhi He, Shuo Chen, Thach-vu Ho and Yang Chai
DOI: 10.1242/dev.196360

**Editor**: Patrick Tam

**Review timeline**

| | |
|---|---|
| Original submission: | 27 August 2020 |
| Editorial decision: | 28 September 2020 |
| First revision received: | 11 November 2020 |
| Accepted: | 7 December 2020 |


---

**Original submission**

First decision letter

MS ID#: DEVELOP/2020/196360

MS TITLE: Ror2-mediated non-canonical Wnt signaling regulates Cdc42 and cell proliferation during tooth root development

AUTHORS: Yuanyuan Ma, Junjun Jing, Jifan Feng, Yuan Yuan, Quan Wen, Xia Han, Jinzhi He, Shuo Chen, Thach-vu Ho, and Yang Chai

I have now received all the referees' reports on the above manuscript, and have reached a decision. The referees' comments are appended below, or you can access them online: please go to BenchPress and click on the 'Manuscripts with Decisions' queue in the Author Area.

As you will see, the referees express considerable interest in your work, but have some significant criticisms and recommend a substantial revision of your manuscript before we can consider publication. If you are able to revise the manuscript along the lines suggested, which may involve further experiments, I will be happy receive a revised version of the manuscript. Your revised paper will be re-reviewed by one or more of the original referees, and acceptance of your manuscript will depend on your addressing satisfactorily the reviewers' major concerns. Please also note that Development will normally permit only one round of major revision.

We are aware that you may be experiencing disruption to the normal running of your lab that make experimental revisions challenging. If it would be helpful, we encourage you to contact us to discuss your revision in greater detail. Please send us a point-by-point response indicating where you are able to address concerns raised (either experimentally or by changes to the text) and where you will not be able to do so within the normal timeframe of a revision. We will then provide further guidance. Please also note that we are happy to extend revision timeframes as necessary.

Please attend to all of the reviewers' comments and ensure that you clearly highlight all changes made in the revised manuscript. Please avoid using 'Tracked changes' in Word files as these are lost in PDF conversion. I should be grateful if you would also provide a point-by-point response detailing how you have dealt with the points raised by the reviewers in the 'Response to Reviewers' box. If you do not agree with any of their criticisms or suggestions please explain clearly why this is so.

© 2020. Published by The Company of Biologists under the terms of the Creative Commons Attribution License (https://creativecommons.org/licenses/by/4.0/).

Reviewer 1

*Advance summary and potential significance to field*

Data obtained on the mechanisms governing tooth root patterning and development are still very limited. The present study by Ma and collaborators clearly shows the involvement of the Ror2/Cdc42/p70S6K axis as one of the signaling pathways underlying tooth root development. This signaling is only active in the tooth mesenchymal part of the root (epithelium not affected) and directly affects the regulation of cell proliferation during root formation.This in turn explains why the root is shorter in the mutants. These data thus contribute to a better understanding of the cellular and molecular mechanisms underlying root morphogenesis. In addition, mutant Ror2 mice display a shortened root size similar to what is observed in a number of patients affected with the Recessive Robinow syndrome. Therefore these data may open the way for potential new avenues for developing therapeutical approaches for root regeneration.

*Comments for the author*

These data are of interest for the readers of Development however before being accepted for publication, a few points listed below should be clarified.
1) Please specify in the manuscript, the number of teeth in the micro-CT analyses and involved in the Cdc-42 activator 1 test (kidney capsule implantations)
2) In Figure 2 D&H the mutant crown appear smaller...
3) Please specify in Mat&Meth how the quantification was performed in Fig2, 3, 7 and S7.
4) In Figure 6 D,H,L, the morphology of the odontoblasts is difficult to see, a higher magnification is required (at least for the paper edition of the journal)
5) How did you determine the concentration of Cdc42 activator-1 to be used in the kidney capsule implantation tests? Did you apply a previously published protocol or did you perform a dose response assay and if yes, could you observe a concentration effect?
3) The mTor appears as important as elF4/p70S6K in the pathway enrichment analysis. However there was no decrease in phosphorylation of mTor detected in the mutants. Could you, in the discusion section, speculate on the possible role of mTor as you have for the Ca signaling pathway also sorted out by the same analysis?
4) In the kidney capsule implantation experiments, you observed that Cdc42 activator 1 treated teeth display "longer" odontoblasts than those of the BSA controls and were of similar size as those in the WT molars. However p7, when investigating Dspp exopression, you observe that dentinogenesis is retarded but that the morphology of the odontoblasts is normal. These two obervations seems a bit contradictory. If odontoblasts are indeed shorter in the Ror2 mutant mice that implies that polarization is likely incomplete (and odontoblasts still immature-which explains the observed lack of/or lower Dspp expression) and all this then suggest that the Ror2/Cdc42 axis acts on mesenchymal cell/odontoblasts differentiation as well as on their proliferation.. Action of RoR2 on terminal differentiation is indeed evoked in the Results section (p 7) but not discussed later. Could you clarify this and maybe speculate on the ROR2/CDC42 potential action on odontoblast differentiation?

Reviewer 2

*Advance summary and potential significance to field*

In this manuscript, Ma and colleagues studied the function of Ror2 during root formation in the mouse molars. Deletion of Ror2 in dental mesenchyme during root formation caused reduced cell proliferation, disrupted the differentiation of odontoblasts and dentinogenesis, and affected the normal development of HERS.
Through RNA Sequencing, the authors identified Cdc42 as a potential downstream mediator of Ror2 signaling during root formation. Ex-vivo culture with activator-soaked beads showed that increased activity of CDC42 can partially restore the reduced proliferation and rescue the shorter length of the root in Osr2-Cre;Ror2fl/fl mutants. In general, the manuscript is well written with nice data that support the hypotheses.

© 2020. Published by The Company of Biologists under the terms of the Creative Commons Attribution License (https://creativecommons.org/licenses/by/4.0/).

*Comments for the author*

1. Is there a general slowing in development in Osr2-Cre;Ror2fl/fl mice? Can the authors provide additional information to stage the animals to make sure both controls and mutants are comparable in their general growth rate?

2. The authors listed differentially expressed candidate genes related to cell proliferation in Fig. 5B. However, there is only one follow-up study: changes in Ccne expression in Osr2-Cre;Ror2fl/fl mice. Can the authors further elaborate on this piece of data?

3. The authors identified Cdc42 mainly by pathway enrichment analysis, which seems indirect. Is there any more direct evidence pointing to Cdc42 as the potential gene responsible for the phenotype shown in Ror2 mutants? For example does Cdc42 show up as one of the candidate genes related to reduction in cell proliferation? What is the expression pattern of Cdc42 in mouse molar? Does it correlate with the proliferating region during molar root formation? What is the change in Cdc42 expression in Osr2-Cre;Ror2fl/fl mutants? Does this expression correlate to that observed in the tooth germs transplanted into the kidney capsules at similar growth stages?

4. Ror2-Cdc42 signaling is also known to mediate changes in the actin cytoskeleton. The involvement of the cytoskeleton has also been suggested earlier via in vitro experiments treated with Cytochalasin D, where a similar morphology was observed (Sohn et al., 2014). It would be interesting to check if proliferation-mediated cytoskeletal changes underlie the altered HERS morphology in the Osr2-Cre;Ror2fl/fl mutants.

5. It is very interesting that the root growth and patterning is regulated via mesenchymal cell proliferation whereas root initiation is under epithelial regulation, via the BMP-Smad-Shh signaling pathway. A loss of Ror2 does not inhibit root initiation. Can the authors comment upon the conservation of this general idea of regulation switching from the epithelium to the mesenchyme which is also seen in tooth development in general?

<u>Reviewer 3</u>

*Advance summary and potential significance to field*

While the development of tooth crown has been studied rather extensively knowledges on the mechanisms regulating the formation of tooth root, an important integral part of a functional tooth, are relatively limited. Previous studies have shown crucial role of canonical Wnt signaling in tooth root formation and patterning, but if non-canonical Wnt signaling is involved in root morphogenesis lacked direct evidence. This study by Ma and colleagues investigated the function of Ror2-mediated non-canonical Wnt signaling in tooth root development. The authors demonstrate that mesenchymal deletion of Ror2 impairs cell proliferation in root mesenchyme, which in turn affects HERS development via tissue-tissue interaction, leading to the formation of shortened roots. Importantly, the authors were able to identify and confirm Cdc42 as a downstream effector of Ror2 mediated signaling in tooth root development. The entire study is well designed and executed. The results are solid and of high quality. This study provides unambiguous evidence supporting a critical role of the non-canonical Wnt signaling in tooth root development.

*Comments for the author*

I have a few comments as below.

1. The difference in cell proliferation rate (Ki67) in dental mesenchyme between control and mutant appears inconsistent in Figure 3 and Figure 7. In Figure 3, the p value is 0.0011 in the percent of Ki67-positive cells between control and mutant. However, in Figure 7, the Ki67-positive cells count around 30% in control with BSA bead and make about 22% in mutant with BSA bead. The p value here would be larger than 0.01. In addition, the PHH4-positive cells count around 4.5% cells in control dental mesenchyme in Figure 3 but make about 13% in control with BSA shown in Figure 7N. Can the authors clarify the difference?

2. In Figure 7M, overall cell proliferation rate (Ki67 labeling) is increased in mutant root mesenchyme with activator treatment, but the increasement is not significant as compared to

© 2020. Published by The Company of Biologists under the terms of the Creative Commons Attribution License (https://creativecommons.org/licenses/by/4.0/).

mutant sample with BSA treatment (p = 0.0204). This is not consistent with the statement made in the text (p10).
3. Significantly impaired cell proliferation was seen in root mesenchyme at PN3.5, but in rescue experiment, tooth germs at PN4.5 were used for transplantation. Can the authors provide rationale?
4. Activator I can increase the activity of Cdc42 robustly within 2-4 hours. It is not clear why the authors conducted cell proliferation assay on rescued samples 3 days after transplantation.
5. I am not sure if it is true result or just accident tissue section selection, it appears to me that Cdc42 activator inhibits cell proliferation in HERS in both control and mutant.
6. It would be very helpful to readers who are interested to follow up the research along this line if the authors can provide details on the 891 DEGs as a supplemental file.
7. It would be interesting to know if incisor root is also affected.
8. Some sentences are difficult to understand. For example, on p8, “Signaling downstream of Ror2 is variable. It has been identified as a regulator of canonical Wnt signaling that affects the stabilization of cytosolic β-catenin in vivo and in vitro”, I had hard time to understand these two sentences.

---

**First revision**

Author response to reviewers' comments

Response to Reviewers’ Comments:

Reviewer 1 Advance Summary and Potential Significance to Field:
Data obtained on the mechanisms governing tooth root patterning and development are still very limited. The present study by Ma and collaborators clearly shows the involvement of the Ror2/Cdc42/p70S6K axis as one of the signaling pathways underlying tooth root development. This signaling is only active in the tooth mesenchymal part of the root (epithelium not affected) and directly affects the regulation of cell proliferation during root formation. This in turn explains why the root is shorter in the mutants. These data thus contribute to a better understanding of the cellular and molecular mechanisms underlying root morphogenesis. In addition, mutant Ror2 mice display a shortened root size similar to what is observed in a number of patients affected with the Recessive Robinow syndrome. Therefore these data may open the way for potential new avenues for developing therapeutical approaches for root regeneration.

Reviewer 1 Comments for the Author:
These data are of interest for the readers of Development however before being accepted for publication, a few points listed below should be clarified.

1. Please specify in the manuscript, the number of teeth in the micro-CT analyses and involved in the Cdc-42 activator 1 test (kidney capsule implantations)
Response: We thank the reviewer for the helpful suggestions. We have labeled the number of teeth/mice in the micro-CT analyses in our manuscript and figure legends, which have been highlighted in red in the revised manuscript.

2. In Figure 2 D&H the mutant crown appear smaller...
Response: We thank the reviewer for the comment. As shown as Fig. 2C, G from the bottom view, the mesiodistal width and buccal-lingual width of the crown in *Ror2* mutant mice appeared comparable to those in the control. Therefore, we carefully compared the 3D images of crown from different sections and calculated the mesiodistal and buccal-lingual dimensions of the crowns at PN12.5 (Response Fig.1A, B, all response figures are at the end of this letter). The results showed that there was no statistically significant difference in either the mesiodistal width or the buccal-lingual width of crowns between *Osr2-Cre;Ror2*$^{fl/fl}$ mice and controls. Taken together, our quantitative analyses confirmed that the overall size of the crown in *Osr2- Cre;Ror2*$^{fl/fl}$ mice was similar to that of the controls.

© 2020. Published by The Company of Biologists under the terms of the Creative Commons Attribution License (https://creativecommons.org/licenses/by/4.0/).

3. Please specify in Mat&Meth how the quantification was performed in Fig2, 3, 7 and S7.
Response: We apologize for the missing information. We have specified the statistical methods in the Materials and Methods section, highlighted in red on pg. 19.

4. In Figure 6 D,H,L, the morphology of the odontoblasts is difficult to see, a higher magnification is required (at least for the paper edition of the journal)
Response: Thank you for this suggestion. We have added higher magnification images to show the morphology of odontoblasts, highlighted with dotted lines in Figure 6D', H', L' and P'.

5. How did you determine the concentration of Cdc42 activator-1 to be used in the kidney capsule implantation tests? Did you apply a previously published protocol or did you perform a dose response assay and if yes, could you observe a concentration effect?
Response: For the rescue study using kidney capsule transplantation, we tested the sustained release of different protein-soaked beads (BSA, activator or protein x) at 37°C *in vitro* (Response Fig. 1C, D, E). The results showed that the concentration of protein released from beads is dynamic and the release rate is different across different proteins. Therefore, it is hard to investigate the concentration effect accurately in kidney capsule transplantation. The concentration of beads-soaked protein in kidney capsule experiments or organ culture has been reported 100 µg/ml according to the previous protocols in different literature (Colnot et al., 2004; Huang et al., 2010; Kratochwil et al.; Wen et al., 2020). The beads soaked with 50 µg/ml activator can sustainably release the protein with the concentration ranged from about 13 to 106 µg/ml (Response Fig. 1C, D, E). Therefore, we used this concentration to soak the beads. The results showed that the activator-soaked beads with 50 µg/ml activator partially rescued the root phenotype in *Osr2-Cre;Ror2*$^{fl/fl}$ molars. Due to time constraints we were unable to test other concentrations.

6. The mTor appears as important as elF4/p70S6K in the pathway enrichment analysis. However there was no decrease in phosphorylation of mTor detected in the mutants. Could you, in the discussion section, speculate on the possible role of mTor as you have for the Ca signaling pathway also sorted out by the same analysis?
Response: Thank you for this question. Because p70S6K is usually regarded as a substrate of mTOR, most genes related to regulation of eIF4 and p70S6K signaling were also categorized as belonging to mTOR signaling by default with the same *p*-value in IPA (Fig. 5B). However, the activation of p70S6K can be dependent or independent of mTOR (Fang et al., 2003; Martin et al., 2014). Therefore, its activation does not necessarily mean that the phosphorylation of mTOR is also changed. In our study, there was no statistical difference in the activation of mTOR between *Ror2* mutant molars and controls (Fig.5C-D). Therefore, it is difficult to speculate about its possible role in Ror2-mediated tooth development.

7. In the kidney capsule implantation experiments, you observed that Cdc42 activator 1 treated teeth display "longer" odontoblasts than those of the BSA controls and were of similar size as those in the WT molars. However p7, when investigating Dspp expression, you observe that dentinogenesis is retarded but that the morphology of the odontoblasts is normal. These two observations seems a bit contradictory. If odontoblasts are indeed shorter in the Ror2 mutant mice that implies that polarization is likely incomplete (and odontoblasts still immature-which explains the observed lack of/or lower Dspp expression) and all this then suggest that the Ror2/Cdc42 axis acts on mesenchymal cell/odontoblasts differentiation as well as on their proliferation.. Action of Ror2 on terminal differentiation is indeed evoked in the Results section (p 7) but not discussed later. Could you clarify this and maybe speculate on the ROR2/CDC42 potential action on odontoblast differentiation?
Response: We also noticed these two different morphologies of odontoblasts after loss of *Ror2* in the dental mesenchyme. We believe that the mechanism underlying the differentiation of odontoblasts is distinct in the apical region of the root (the elongation region of the root) from the furcation region because the developmental modes are quite different across these two regions. For example, Bae. et al. classified odontoblasts in the apical region of mouse molars into three different types: preodontoblasts, odontoblasts and a new population they named "apical odontoblasts" (AOds) (Bae et al., 2013). They argued that AOds are a transitional form in the transition from preodontoblasts to odontoblasts and are the principal cells for root elongation. However, AOds have not been reported in the furcation region. To take another example, *Osx*$^{Col}$ and *Osx*$^{OC}$ mice have been reported to form short roots with a translucent furcation area due to

© 2020. Published by The Company of Biologists under the terms of the Creative Commons Attribution License (https://creativecommons.org/licenses/by/4.0/). 

extremely thin interradicular dentin (Kim et al., 2015). This site-specific defect may be associated with the intrinsic differences between the development of the elongation region and the furcation region.
We double-checked the morphology of odontoblasts in the apical region in both control and Ror2 mutant mice and confirmed that the morphology of odontoblasts and preodontoblasts in *Ror2* mutant mice showed similar morphology to those of the controls (Fig. 4G,H). The only difference is that the transitional form of odontoblasts in the control molars showed a condensed, low columnar alignment, but a looser distribution of odontoblasts in *Osr2-Cre;Ror2$^{fl/fl}$* molars (Fig. 4G, H). We are appreciative of this question, which has helped us to make the description more accurate. We also discuss cell differentiation in the manuscript on pg. 13.

Reviewer 2 Advance Summary and Potential Significance to Field:
In this manuscript, Ma and colleagues studied the function of Ror2 during root formation in the mouse molars. Deletion of Ror2 in dental mesenchyme during root formation caused reduced cell proliferation, disrupted the differentiation of odontoblasts and dentinogenesis, and affected the normal development of HERS. Through RNA Sequencing, the authors identified Cdc42 as a potential downstream mediator of Ror2 signaling during root formation. Ex-vivo culture with activator-soaked beads showed that increased activity of CDC42 can partially restore the reduced proliferation and rescue the shorter length of the root in Osr2-Cre;Ror2fl/fl mutants. In general, the manuscript is well written with nice data that support the hypotheses.

Reviewer 2 Comments for the Author:
1. Is there a general slowing in development in *Osr2-Cre;Ror2$^{fl/fl}$* mice? Can the authors provide additional information to stage the animals to make sure both controls and mutants are comparable in their general growth rate?
Response: Thank you for this question. Although we observed that *Osr2-Cre;Ror2$^{fl/fl}$* mice have a smaller body size, it is difficult to reach the conclusion that there is a general slowing of development in *Ror2* mutant mice. We carefully examined both control and Ror2 mutant mice and compared them according to the description of developmental stages provided by Jackson Laboratory. At PN3.5, the ear flap in both control and Ror2 mutant mice is starting to come away from the head and the skin color is darkening in both groups (Response Fig. 2A at the end of this letter). At PN9.5, the fur and the skin color in *Osr2-Cre;Ror2$^{fl/fl}$* mice are almost the same as those of the control (Response Fig. 2B). Teeth typically begin to erupt at PN11 in wild type mice. However, we found that the incisors begin to erupt at PN9.5 in some *Osr2-Cre;Ror2$^{fl/fl}$* mice (Response Fig. 2C). Therefore, we think that there is no general slowing of development in *Osr2-Cre;Ror2$^{fl/fl}$* mice. Due to the cellular defects observed at PN3.5, we performed further measurements to ensure that the development of the mouse molars is comparable at this stage between controls and Ror2 mutants. We calculated the surface area and volume of the molars and found no statistical difference between the control and *Ror2* mutant mice (Response Fig. 2D- G). We also found that the differentiation of odontoblasts was unchanged after the deletion of *Ror2* in the dental mesenchyme at PN3.5 (Response Fig. 2H-K).

2. The authors listed differentially expressed candidate genes related to cell proliferation in Fig. 5B. However, there is only one follow-up study: changes in *Ccne1* expression in *Osr2-Cre;Ror2$^{fl/fl}$* mice. Can the authors further elaborate on this piece of data?
Response: Thank you for your suggestion. We have also tested the expression of other cell proliferation-related genes including *Ccna2, Ect2, Ccnc* and *Ccng1* as shown in Table. S2. *Ccna2* is well accepted as a key cell-cycle regulator (S phase) and *Ect2* has the potential to regulate cell mitosis. *In situ* hybridization showed that the expression of *Ccna2* and *Ect2* was present in the apical region of the mouse molar but the expression levels of these genes were significantly decreased in *Osr2-Cre;Ror2$^{fl/fl}$* mice compared to the controls (Fig. 5N-R, Fig. S8D-H). This is consistent with the results of RNA-sequencing (Table. S2). We have added this data into the manuscript. Although *Ccng1* and *Ccnc* also belong to the cyclin family of proteins, it is not clear how they may function in cell cycle progression. Based on their expression patterns in mouse molars (Response Fig. 2L-O), we speculate they may have less of an effect on cell proliferation in tooth root development.

3. The authors identified Cdc42 mainly by pathway enrichment analysis, which seems indirect. Is there any more direct evidence pointing to Cdc42 as the potential gene responsible for the phenotype shown in Ror2 mutants? For example, does Cdc42 show up as one of the candidate

© 2020. Published by The Company of Biologists under the terms of the Creative Commons Attribution License (https://creativecommons.org/licenses/by/4.0/).

genes related to reduction in cell proliferation? What is the expression pattern of Cdc42 in mouse molar? Does it correlate with the proliferating region during molar root formation? What is the change in Cdc42 expression in Osr2-Cre;Ror2fl/fl mutants? Does this expression correlate to that observed in the tooth germs transplanted into the kidney capsules at similar growth stages?

Response: We found that *Cdc42* mRNA expression was slightly decreased in the RNA- sequencing data, but *Cdc42* was not included in the 891 DEGs. This is likely due to the fact that the change in Cdc42 mRNA level is subtle. We detected no change in the total Cdc42 protein level (Fig. 5C). The function of Cdc42 is typically dependent on its protein activation (GTP- Cdc42). Therefore, we focused on its activation rather than its gene expression level in the manuscript. In order to further investigate the function of Cdc42 in cell proliferation, we harvested the apical half of the dental mesenchyme from wild type mice at PN3.5 for cultivation *in vitro*. The cells treated with ZCL278, a Cdc42 GTPase inhibitor, showed a decrease in cell mitosis (Supplementary Fig. S8A, B, C). This indicates that the activation of Cdc42 is related to the proliferation of the dental mesenchyme.

We agree that the expression pattern of *Cdc42* in the mouse molar is important for the phenotype observed in *Ror2* mutant mice. Therefore, we did *in situ* hybridization for *Cdc42* and immunostaining for Ki67/Ror2. These results showed that there is an overlap in the expression of *Cdc42* and Ki67 in the apical region of mouse molar at PN3.5, suggesting its correlation with cell proliferation during root growth (Fig. 5F, G). Overlap was also observed between the expression of *Cdc42* and Ror2 (Fig. 5H). For kidney capsule transplantation, we detected *Cdc42* expression in the apical region of mouse molar 3 days after transplantation (at PN4.5), which was similar to its expression *in vivo* at PN7.5 (Supplementary Fig. S8I-L).

4. Ror2-Cdc42 signaling is also known to mediate changes in the actin cytoskeleton. The involvement of the cytoskeleton has also been suggested earlier via in vitro experiments treated with Cytochalasin D, where a similar morphology was observed (Sohn et al., 2014). It would be interesting to check if proliferation-mediated cytoskeletal changes underlie the altered HERS morphology in the *Osr2-Cre;Ror2$^{fl/fl}$* mutants.

Response: Thank you for your suggestion. We were indeed inspired by this publication (Sohn et al., 2014). Ror2/Cdc42 signaling plays an important role in cytoskeletal morphology. However, it was also reported that no abnormalities in the cytoskeletal morphology were found in *Cdc42*- deficient migrating neural crest stem cells (Fuchs et al., 2009). We tested the distribution of phalloidin in the apical region of mouse molars *in vivo* and in dental mesenchymal explants *in vitro* from control and *Ror2* mutant molars (Response Fig. 3A-F). There was no obvious change in the expression of phalloidin in either the epithelium or mesenchyme in the apical region *in vivo* (Response Fig. 3A, A’, D, D’). *In vitro*, cells can normally migrate from the tissue explants with similar phalloidin distribution in both controls and *Ror2* mutants (Response Fig. 3B-F). But under higher magnification, cells from *Ror2* mutant mice showed shorter filopodia on the leading edge than those of the control (Response Fig. 3C’, F’). Based on these limited results, it is still difficult to determine whether there is a change in the cytoskeleton. Furthermore, there is no direct evidence to show which structure of the cytoskeleton plays a role in root development or how the mesenchymal cytoskeleton affects root elongation. We agree that in the future it would be interesting to explore the significance of the cytoskeleton and the relationship between cell proliferation and the cytoskeleton during root development.

5. It is very interesting that the root growth and patterning is regulated via mesenchymal cell proliferation whereas root initiation is under epithelial regulation, via the BMP-Smad-Shh signaling pathway. A loss of Ror2 does not inhibit root initiation. Can the authors comment upon the conservation of this general idea of regulation switching from the epithelium to the mesenchyme, which is also seen in tooth development in general?

Response: This is indeed a very interesting point. First, we also believe that the mesenchymal signaling plays a crucial role in root number and root length based on published studies. For example, the deletion of genes such as *Smad4*, *Tgfbr2* and *Bmp4* in the dental mesenchyme leads to shortened roots in mouse molars (Li et al., 2017). Although root development is impaired in these models, the roots are present, albeit shortened. This suggests that these signaling molecules in the dental mesenchyme may be related to root elongation rather than root initiation. It has

© 2020. Published by The Company of Biologists under the terms of the Creative Commons Attribution License (https://creativecommons.org/licenses/by/4.0/).

also been reported that Ezh2 in the dental mesenchyme regulates root patterning and furcation formation (Jing et al., 2019). Therefore, the dental mesenchyme is important for root patterning and root morphogenesis, and mesenchymal proliferation is one of the contributors to root formation. Second, epithelial regulation of root initiation has also been demonstrated in *K14-Cre;Smad4$^{fl/fl}$* mice, which exhibit no root formation (Huang et al., 2010). In *K14-rtTA;Teto-Cre;Smad4$^{fl/fl}$* mice, there is still formation of a shortened root (Li et al., 2015). Considering these two models, we think Smad4 regulates root initiation at early stages but modulates root elongation at a later stage. Although HERS is critical for root initiation, we cannot exclude the importance of the dental mesenchyme in root initiation. For example, no molar roots form in *OC-Cre:Ctnnb1$^{CO/CO}$* mice or *Osr2-Cre;Fgf10$^{Tg}$* mice (Kim et al., 2013; Li et al., 2017). In particular, in *OC-Cre:Ctnnb1$^{CO/CO}$* mice, roots fail to form due to the disruption of apical odontoblast differentiation despite no distinguishable difference in the initial extension of HERS. This suggests that Wnt/ß-catenin and Fgf signaling in the dental mesenchyme may play a key role in the initiation of root formation. Third, tooth development begins with crown formation during the embryonic stage. The first signaling centers appear in the dental placodes, followed by the enamel knot signaling center at the bud-to-cap transition. The odontogenic mesenchyme is induced by BMP and FGF signaling from the dental epithelium. Therefore, at the early stage of tooth development, signaling from the dental epithelium plays a leading role. However, based on the reported mouse models, it seems that the signaling from the dental mesenchyme becomes more important during postnatal tooth root development.

Reviewer 3 Advance Summary and Potential Significance to Field:
While the development of tooth crown has been studied rather extensively, knowledges on the mechanisms regulating the formation of tooth root, an important integral part of a functional tooth, are relatively limited. Previous studies have shown crucial role of canonical Wnt signaling in tooth root formation and patterning, but if non-canonical Wnt signaling is involved in root morphogenesis lacked direct evidence. This study by Ma and colleagues investigated the function of Ror2-mediated non-canonical Wnt signaling in tooth root development. The authors demonstrate that mesenchymal deletion of Ror2 impairs cell proliferation in root mesenchyme, which in turn affects HERS development via tissue-tissue interaction, leading to the formation of shortened roots. Importantly, the authors were able to identify and confirm Cdc42 as a downstream effector of Ror2 mediated signaling in tooth root development. The entire study is well designed and executed. The results are solid and of high quality. This study provides unambiguous evidence supporting a critical role of the non-canonical Wnt signaling in tooth root development.

Reviewer 3 Comments for the Author: I have a few comments as below.

1. The difference in cell proliferation rate (Ki67) in dental mesenchyme between control and mutant appears inconsistent in Figure 3 and Figure 7. In Figure 3, the p value is 0.0011 in the percent of Ki67-positive cells between control and mutant. However, in Figure 7, the Ki67-positive cells count around 30% in control with BSA bead and make about 22% in mutant with BSA bead. The p value here would be larger than 0.01. In addition, the PHH4-positive cells count around 4.5% cells in control dental mesenchyme in Figure 3 but make about 13% in control with BSA shown in Figure 7N. Can the authors clarify the difference?

Response: Thank you for this question. There are two reasons why the *p*-value and cell count in Fig. 3 are different from those in Fig. 7. First, in Fig. 3, we detected the cell proliferation at PN3.5 *in vivo*. However, in Fig. 7, we detected Ki67-positive cells 3 days after transplantation beginning at PN4.5. The second reason is that we used different statistical methods in our *in vivo* and *ex vivo* study. We have now made this clear in the Materials and Methods section in the revised manuscript. *In vivo*, statistical comparisons were performed using Student's *t* test. For *ex vivo* study, statistical significance was determined using two-way analysis of variance (ANOVA) followed by Tukey's multiple comparison tests. We have increased the N number in all groups to 5 samples for cell proliferation analysis in our *ex vivo* study. However, the *p*-value is still larger than it is for the *in vivo* data in Fig. 3. We think this is reasonable because two-way ANOVA takes the variation of all data from all four experimental groups into consideration. Therefore, the *p*-value is different from that generated by Student's *t* test.

2. In Figure 7M, overall cell proliferation rate (Ki67 labeling) is increased in mutant root mesenchyme with activator treatment, but the increasement is not significant as compared to

© 2020. Published by The Company of Biologists under the terms of the Creative Commons Attribution License (https://creativecommons.org/licenses/by/4.0/).

mutant sample with BSA treatment (p = 0.0204). This is not consistent with the statement made in the text (p10).

Response: Thank you for your suggestion. We have increased the N number in all groups to 5 samples for statistical analysis and revised the manuscript accordingly.

3. Significantly impaired cell proliferation was seen in root mesenchyme at PN3.5, but in rescue experiment, tooth germs at PN4.5 were used for transplantation. Can the authors provide rationale?

Response: We transplanted tooth germs into kidney capsule at PN4.5 because of the technical difficulty of performing this technique with tissue from younger mice. We have tried to transplant tooth germs at PN0 and PN2.5. However, we have found it is prohibitively difficult to hold the tooth germ using instruments because it is very soft and easy to sustain damage during the process. At PN4.5, it is easy to hold the tooth germs and transplant them into the kidney capsule. Furthermore, the cellular defect at PN4.5 was almost the same as that at PN3.5 (Response Fig. 4A-B). Therefore, we decided to use tooth germs at PN4.5.

4. Activator I can increase the activity of Cdc42 robustly within 2-4 hours. It is not clear why the authors conducted cell proliferation assay on rescued samples 3 days after transplantation.

Response: We apologize that the information about the action time of Cdc42 activator in medium is not appropriate here for kidney capsule transplantation. We have revised our manuscript on pg. 10. As shown in Response Fig. 1C-E, the release of protein from beads takes about 8-18 hrs. The protein also takes time to penetrate into the tissue to trigger the signal transduction, alter gene expression and show changes at the cellular level. Huang et al. detected the rescue of gene expression after 2 days of implanting beads into the dental mesenchyme in organ culture in BGJB culture medium (Huang et al., 2010). Therefore, we tested whether the cellular defect was rescued after 3 days transplantation.

5. I am not sure if it is true result or just accident tissue section selection, it appears to me that Cdc42 activator inhibits cell proliferation in HERS in both control and mutant.

Response: Thank you for your kind reminder. We have checked different sections in our samples and replaced the pictures using more representative sections from the samples. After careful evaluation we concluded there was no change in cell proliferation in HERS of control or *Ror2* mutant tooth germs.

6. It would be very helpful to readers who are interested to follow up the research along this line if the authors can provide details on the 891 DEGs as a supplemental file.

Response: Thank you for your suggestion. We now show 15 changed genes generated from RNA-sequencing in Table S2 and have provided the full list of 891 genes in Table S3. RNA-sequencing data will be submitted to GEO and FaceBase.

7. It would be interesting to know if incisor root is also affected.

Response: We collected samples at postnatal 1 month for incisor analysis. MicroCT images showed that the incisors of *Ror2* mutant mice had decreased in length, as shown in Response Fig. 4C-E. Therefore, we think that mesenchymal *Ror2* may also play a role in incisor development.

8. Some sentences are difficult to understand. For example, on p8, “Signaling downstream of Ror2 is variable. It has been identified as a regulator of canonical Wnt signaling that affects the stabilization of cytosolic β-catenin in vivo and in vitro”, I had hard time to understand these two sentences.
Response: We apologize for this. We have revised these sentences in the manuscript on pg. 8.

We greatly appreciate the editor and reviewers for these helpful comments, which have helped to improve our study.

© 2020. Published by The Company of Biologists under the terms of the Creative Commons Attribution License (https://creativecommons.org/licenses/by/4.0/).

References:

**Bae, C.-H., Kim, T.-H., Chu, J.-Y. and Cho, E.-S.** (2013). New population of odontoblasts responsible for tooth root formation. *Gene Expression Patterns* **13**, 197-202.
**Colnot, C., Lu, C., Hu, D. and Helms, J. A.** (2004). Distinguishing the contributions of the perichondrium, cartilage, and vascular endothelium to skeletal development. *Developmental Biology* **269**, 55-69.
**Fang, Y., Park, I.-H., Wu, A.-L., Du, G., Huang, P., Frohman, M. A., Walker, S. J., Brown, H. A. and Chen, J.** (2003). PLD1 Regulates mTOR Signaling and Mediates Cdc42 Activation of S6K1. *Current Biology* **13**, 2037-2044.
**Fuchs, S., Herzog, D., Sumara, G., Büchmann-Møller, S., Civenni, G., Wu, X., Chrostek-Grashoff, A., Suter, U., Ricci, R., Relvas, J. B., et al.** (2009). Stage-Specific Control of Neural Crest Stem Cell Proliferation by the Small Rho GTPases Cdc42 and Rac1. *Cell stem cell* **4**, 236-247.
**Huang, X., Xu, X., Bringas, P., Jr., Hung, Y. P. and Chai, Y.** (2010). Smad4-Shh-Nfic signaling cascade-mediated epithelial-mesenchymal interaction is crucial in regulating tooth root development. *Journal of bone and mineral research : the official journal of the American Society for Bone and Mineral Research* **25**, 1167-1178.
**Jing, J., Feng, J., Li, J., Han, X., He, J., Ho, T.-V., Du, J., Zhou, X., Urata, M. and Chai, Y.** (2019). Antagonistic interaction between Ezh2 and Arid1a coordinates root patterning and development via Cdkn2a in mouse molars. *Elife* **8**, e46426.
**Kim, T. H., Bae, C. H., Lee, J. C., Kim, J. E., Yang, X., de Crombrugghe, B. and Cho, E. S.** (2015). Osterix regulates tooth root formation in a site-specific manner. *Journal of dental research* **94**, 430-438.
**Kim, T. H., Bae, C. H., Lee, J. C., Ko, S. O., Yang, X., Jiang, R. and Cho, E. S.** (2013). beta-catenin is required in odontoblasts for tooth root formation. *Journal of Dental Research* **92**, 215-221.
**Kratochwil, K., Galceran J Fau - Tontsch, S., Tontsch S Fau - Roth, W., Roth W Fau - Grosschedl, R. and Grosschedl, R.** FGF4, a direct target of LEF1 and Wnt signaling, can rescue the arrest of tooth organogenesis in Lef1(-/-) mice.
**Li, J., Feng, J., Liu, Y., Ho, T.-V., Grimes, W., Ho, H. A., Park, S., Wang, S. and Chai, Y.** (2015). BMP-SHH signaling network controls epithelial stem cell fate via regulation of its niche in the developing tooth. *Developmental cell* **33**, 125-135.
**Li, J., Parada, C. and Chai, Y.** (2017). Cellular and molecular mechanisms of tooth root development. *Development (Cambridge, England)* **144**, 374-384.
**Martin, T. D., Dennis, M. D., Gordon, B. S., Kimball, S. R. and Jefferson, L. S.** (2014). mTORC1 and JNK coordinate phosphorylation of the p70S6K1 autoinhibitory domain in skeletal muscle following functional overloading. *Am J Physiol Endocrinol Metab.* **306**, E1397-1405.
**Wen, Q., Jing, J., Han, X., Feng, J., Yuan, Y., Ma, Y., Chen, S., Ho, T.-V. and Chai, Y.** (2020). Runx2 Regulates Mouse Tooth Root Development Via Activation of WNT Inhibitor NOTUM. *Journal of Bone and Mineral Research* **n/a**.

© 2020. Published by The Company of Biologists under the terms of the Creative Commons Attribution License (https://creativecommons.org/licenses/by/4.0/).

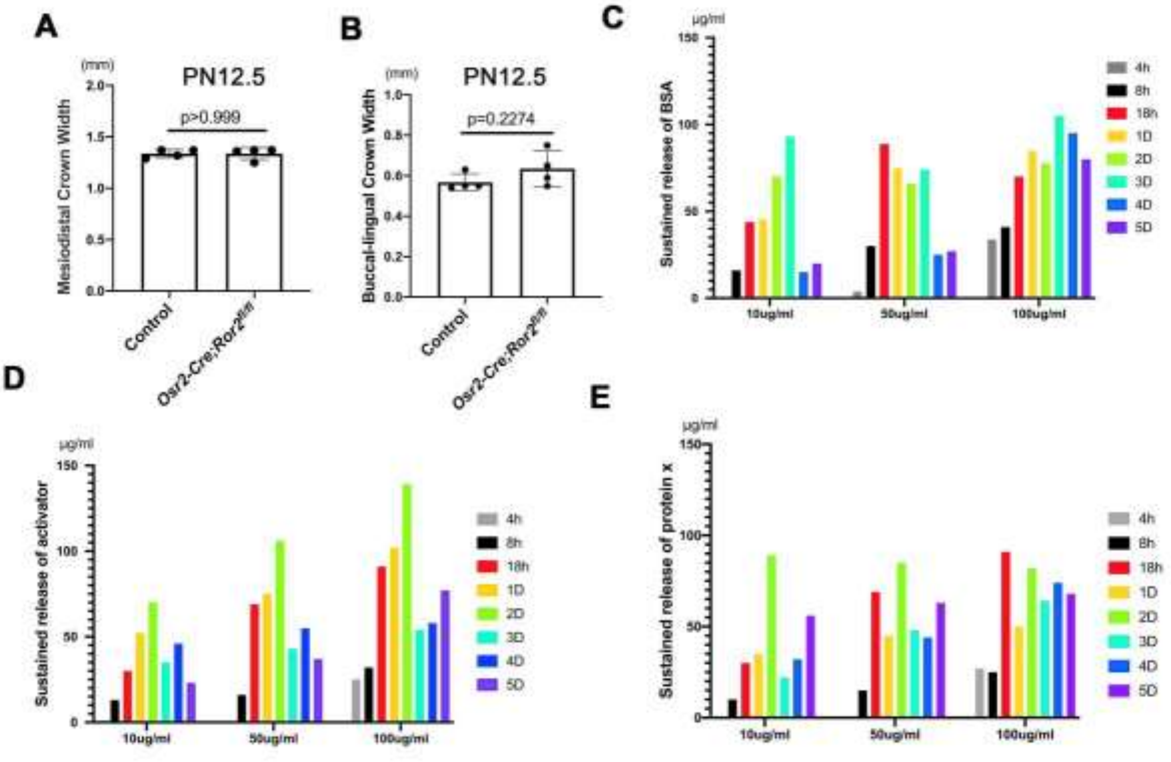


Response Fig. 1

© 2020. Published by The Company of Biologists under the terms of the Creative Commons Attribution License (https://creativecommons.org/licenses/by/4.0/).

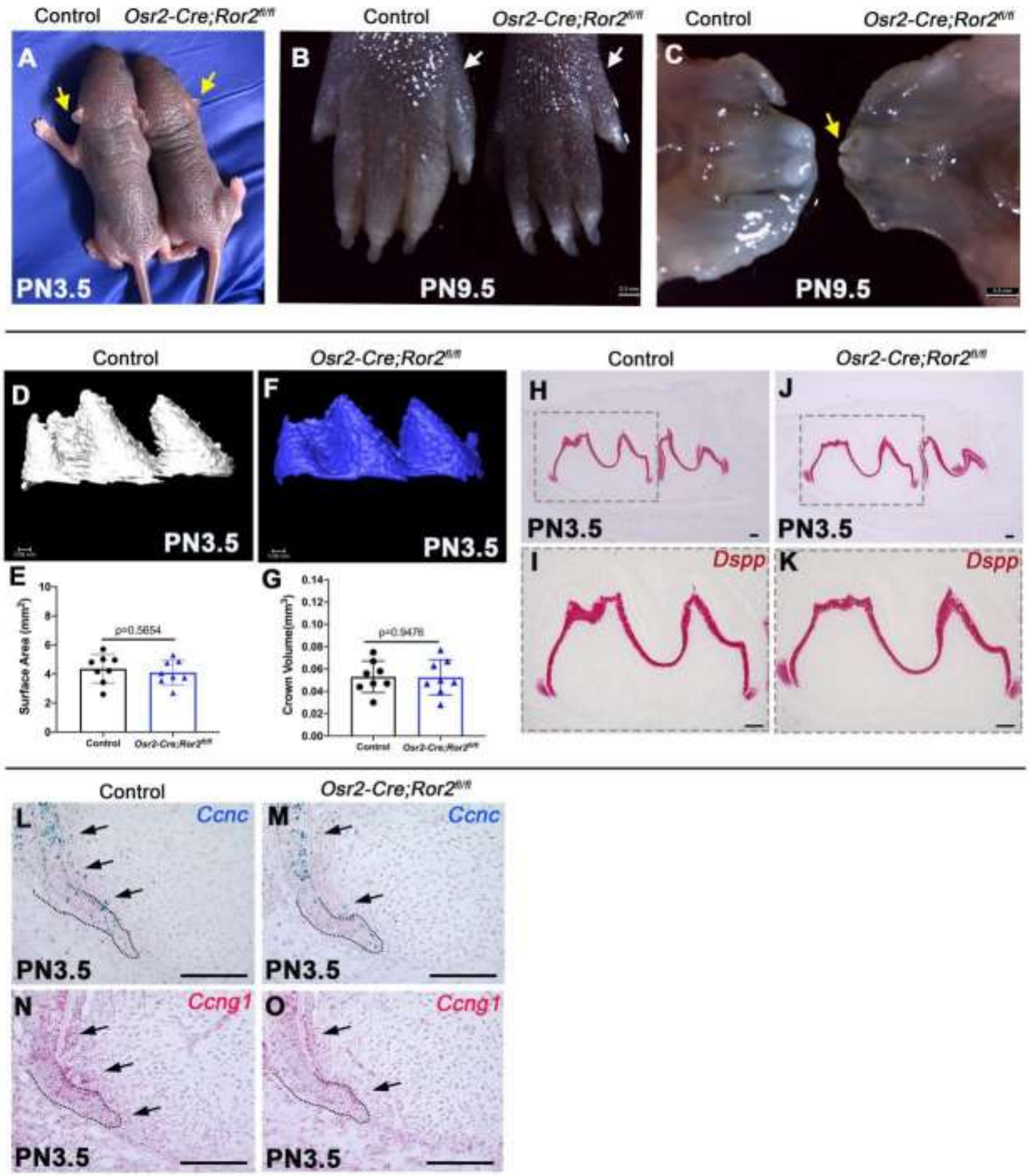


Response Fig. 2

© 2020. Published by The Company of Biologists under the terms of the Creative Commons Attribution License (https://creativecommons.org/licenses/by/4.0/).

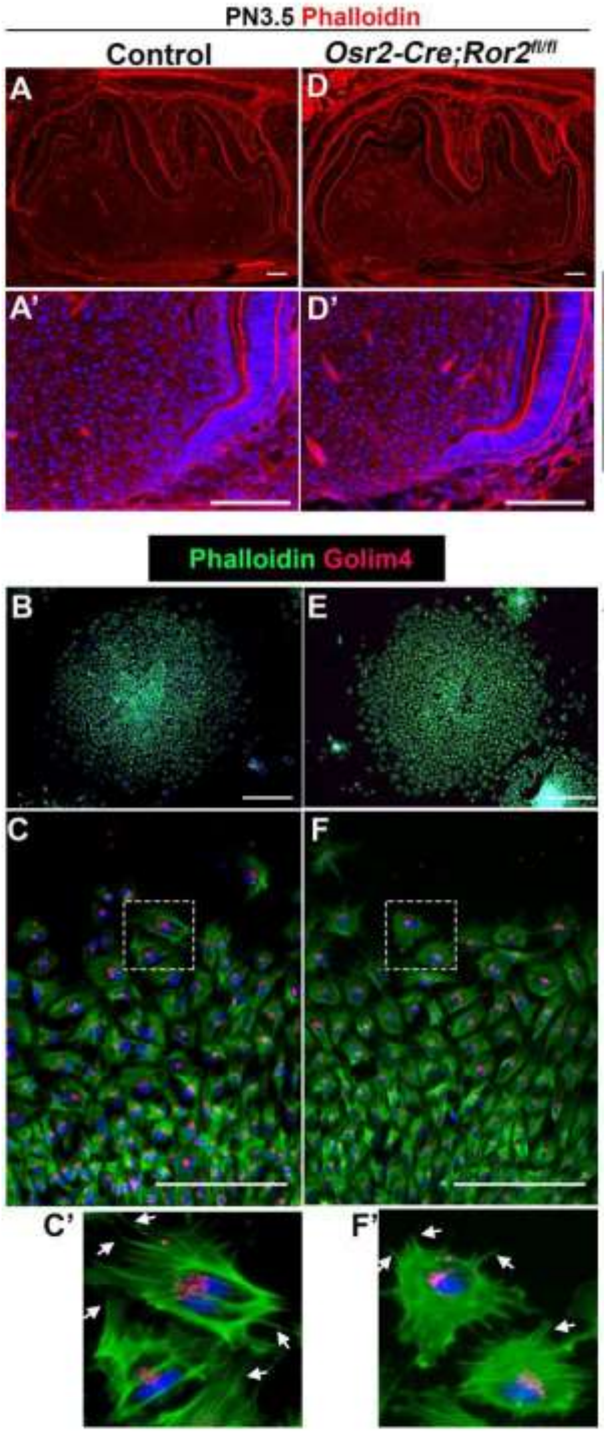


Response Fig. 3

© 2020. Published by The Company of Biologists under the terms of the Creative Commons Attribution License (https://creativecommons.org/licenses/by/4.0/).

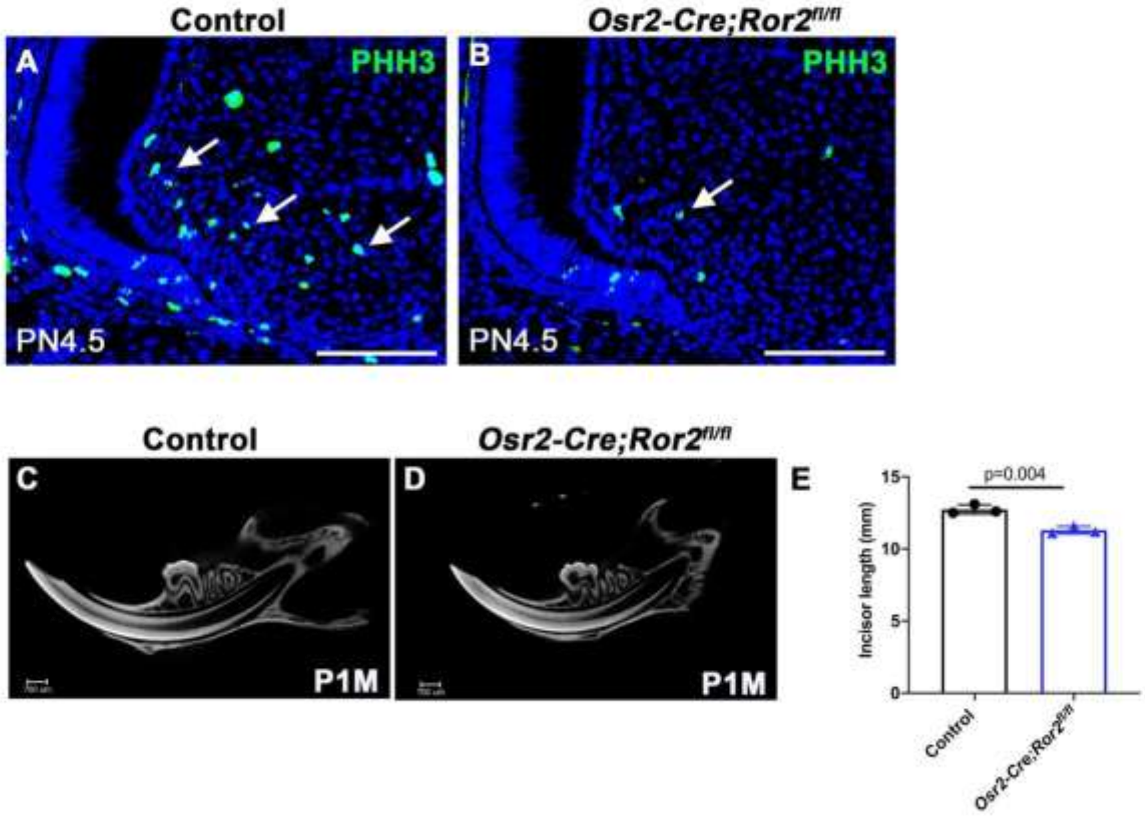


Response Fig. 4

---

Second decision letter

MS ID#: DEVELOP/2020/196360

MS TITLE: Ror2-mediated non-canonical Wnt signaling regulates Cdc42 and cell proliferation during tooth root development

AUTHORS: Yuanyuan Ma, Junjun Jing, Jifan Feng, Yuan Yuan, Quan Wen, Xia Han, Jinzhi He, Shuo Chen, Thach-vu Ho, and Yang Chai
ARTICLE TYPE: Research Article

I am happy to tell you that your manuscript has been accepted for publication in Development, pending our standard ethics checks.

Reviewer 1

*Advance summary and potential significance to field*

Data obtained on the mechanisms governing tooth root patterning and development are still very limited. The present study by Ma and collaborators clearly shows the involvement of the Ror2/Cdc42/p70S6K axis as one of the signaling pathways underlying tooth root development. This signaling is only active in the tooth mesenchymal part of the root (epithelium not affected) and directly affects the regulation of cell proliferation during root formation.This in turn explains why the root is shorter in the mutants. These data thus contribute to a better understanding of the cellular and molecular mechanisms underlying root morphogenesis. In addition, mutant Ror2 mice

© 2020. Published by The Company of Biologists under the terms of the Creative Commons Attribution License (https://creativecommons.org/licenses/by/4.0/). 

display a shortened root size similar to what is observed in a number of patients affected with the Recessive Robinow syndrome. Therefore these data may open the way for potential new avenues for developing therapeutical approaches for root regeneration.

*Comments for the author*

I am satisfied by the answers to my questions and have no further comments

Reviewer 2

*Advance summary and potential significance to field*

The authors have addressed my previous concerns adequately.

*Comments for the author*

The authors have addressed my previous concerns adequately.

Reviewer 3

*Advance summary and potential significance to field*

The authors have satisfactorily addressed the concerns and questions raised on the previous submission. I do not have further comments.

*Comments for the author*

Concerns and questions have been nicely addressed. No further comments.

© 2020. Published by The Company of Biologists under the terms of the Creative Commons Attribution License (https://creativecommons.org/licenses/by/4.0/).
